# Primary human intestinal organoids model enteric infection of monkeypox virus and enable scalable drug discovery

**DOI:** 10.1126/sciadv.aea8280

**Published:** 2026-03-27

**Authors:** Pengfei Li, Xin Wang, Jiangrong Zhou, Yang Yao, Yining Wang, Guige Xu, Rick Schraauwen, Ana Maria Gonçalves da Silva, Charlotte de Henau, Roberto Incitti, Dewy Mae Offermans, Annemarie C. de Vries, Denis E. Kainov, Intikhab Alam, Karine Raymond, Amaro Nunes Duarte-Neto, Marcel J. C. Bijvelds, Qiuwei Pan

**Affiliations:** ^1^ Precision Medicine Translational Research Center, West China Hospital, Sichuan University, Chengdu 610041, China.; ^2^ Department of Gastroenterology and Hepatology, Erasmus MC-University Medical Center, Rotterdam, Netherlands.; ^3^ State Key Laboratory of Oral Diseases & National Center for Stomatology & National Clinical Research Center for Oral Diseases, Department of Implantology, West China Hospital of Stomatology, Sichuan University, Chengdu, Sichuan 610041, China.; ^4^ Department of Pathology, Erasmus MC-University Medical Center, Rotterdam, Netherlands.; ^5^ Departamento de Patologia, Faculdade de Medicina, Universidade de São Paulo, São Paulo, SP, Brazil.; ^6^ Department of Anatomy and Embryology, Leiden University Medical Center, Leiden, Netherlands.; ^7^ The Novo Nordisk Foundation Center for Stem Cell Medicine (reNEW), Leiden University Medical Center, Leiden, Netherlands.; ^8^ Computational Bioscience Research Center, King Abdullah University of Science and Technology, Thuwal, Saudi Arabia.; ^9^ Department of Clinical and Molecular Medicine, Norwegian University of Science and Technology, 7028 Trondheim, Norway.; ^10^ University of Grenoble Alpes, CEA, Inserm, IRIG, UA13 BGE, Biomics, Grenoble, France.

## Abstract

Monkeypox virus (MPXV) infection-associated intestinal manifestations, including diarrhea and proctitis, have been frequently reported during mpox outbreaks. Here, we present clinical evidence that MPXV can directly infect the human intestine and induce lesions. Intriguingly, primary organoids cultured from human ileum and rectum support productive infections by MPXV strains from clade IIb, Ia, and Ib, which are responsible for the 2022–2023 global outbreak and concurrent outbreaks in Africa. Given that primary intestinal organoids can be rapidly expanded at large scale, we were able to screen a broad-spectrum antiviral drug library. We identified 12 leading candidates of safe-in-human agents, including clinically used drugs such as clofarabine. We extensively validated the anti-MPXV activity of clofarabine in human intestinal and skin organoids, consistently demonstrating potent antiviral activity against clade Ia, Ib, and IIb strains. These findings are important for better understanding the clinical manifestations of mpox. Primary intestinal organoid-based infection models and the established antiviral drug discovery pipeline bear major implications for responding to the current mpox global health emergency and sustaining epidemic poxvirus preparedness.

## Teaser

Human intestinal organoids model enteric infection of monkeypox virus and enable rapid screening of antiviral drugs.

## INTRODUCTION

The monkeypox virus (MPXV) belongs to the *Orthopoxvirus* genus of the family *Poxviridae*. MPXV has a linear double-stranded DNA genome of ~200 kb, encoding ~200 viral proteins (*1*). MPXV comprises two distinct clades: clade I, which includes subclades Ia and Ib, and clade II, which includes subclades IIa and IIb. The clade IIb strain caused the 2022–2023 global mpox outbreak (*2*), whereas clade Ia and Ib strains are responsible for concurrent outbreaks in Africa (*3*, *4*). The World Health Organization has declared a Public Health Emergency of International Concern twice—once for the 2022–2023 global mpox outbreak and once for the concurrent outbreaks in Africa (*5*).

Although skin lesions are the most classical symptom, MPXV infection can cause a broad spectrum of systemic manifestations, such as diarrhea, liver injury, myopericarditis, acute kidney injury, and respiratory complications (*6*–*8*). Notably, proctitis—an inflammatory disorder of the rectum—emerged as a new clinical presentation during the 2022–2023 global mpox outbreak, with an incidence rate of ~20% (*9*, *10*). In mpox patients with gastrointestinal manifestations, proctitis and symptoms such as rectal pain, diarrhea, and vomiting are prevalent (*11*). Systemic manifestations are often associated with worse clinical outcomes (*8*, *9*), yet these atypical presentations of mpox remain poorly studied.

Patients with severe mpox require hospitalization, supportive care, and antiviral treatment, but no approved medication is currently available specifically for treating MPXV infection. The antiviral drug tecovirimat was approved for treating smallpox under the US Food and Drug Administration’s (FDA’s) Animal Rule, based on its efficacy in relevant animal models using related orthopoxviruses, including MPXV (*12*). It blocks the final steps of virus maturation and release from infected cells by disrupting the major envelope-wrapping protein VP37 (*13*). Tecovirimat has been widely prescribed under compassionate use for treating clade IIb MPXV infection during the global outbreak (*14*). However, results from two recently conducted randomized, placebo-controlled trials, showed no clinical benefit of tecovirimat treatment for children and adults infected with clade I MPXV (*15*) or adults infected with clade II MPXV (*16*, *17*). Cidofovir and its prodrug brincidofovir, which function as viral DNA polymerase inhibitors (*13*), have also been occasionally prescribed for treating mpox, but their clinical efficacy remains undefined (*17*, *18*).

Given the common prevalence of mpox-associated gastrointestinal manifestations, we first examined clinical evidence and found that MPXV can directly infect the human intestine and cause numerous lesions. Next, we demonstrated that primary organoids derived from human small intestine and rectal tissues support productive infection by both clade I and II MPXV isolates. The infection triggered robust virus-host interactions. Because primary intestinal organoids can be expanded at large scale, we conducted antiviral drug screening and identified potent inhibitors of MPXV infection.

## RESULTS

### Clade IIb MPXV infection in human intestine and cultured primary intestinal organoids

**Fig. 1.**
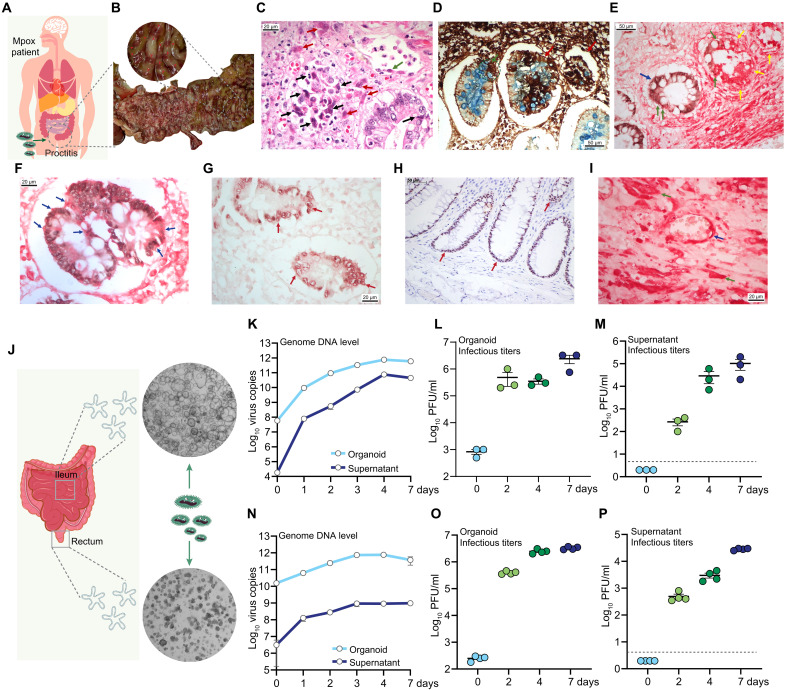
Clade IIb MPXV infection in human intestine and cultured primary intestinal organoids. (**A**) Schematic of MPXV infection in human colon created using Adobe Illustrator. (**B**) Lesions in descending colon (red arrows). (**C**) H&E staining of infected colon: black arrows show shrunken eosinophilic cytoplasm and condensed chromatin; red arrows show Guarnieri-like inclusions; green arrow shows inclusion in an endothelial cell. (**D**) IHC with antivaccinia (MPXV detection) and Alcian blue (mucus visualization): green arrow marks gland with partial antigen staining; red arrows mark glands with extensive staining. (**E**) IHC with anti-vaccinia (red) and anti-CDX2 (brown) in ulcerative mucosa: blue arrow shows epithelial cells with dense nuclei and vacuolated cytoplasm; green arrows show apoptotic cells with pycnotic nuclei; yellow arrows show MPXV-positive necrotic cells. (**F**) Magnified IHC image: blue arrows indicate reactive epithelium with nuclear density and vacuolated cytoplasm. (**G**) IHC in mucosa distant from ulcers: red arrows show epithelium with nuclear density and lamina propria edema; CDX2 positive, MPXV negative. (**H**) IHC in normal tissue: CDX2 positive (red arrow), MPXV negative. (**I**) IHC near peritoneum: blue arrow shows endothelial cells in small vessel; green arrows show MPXV infection in interstitial cells. (**J**) Schematic of primary intestinal organoid isolation. (**K**) Viral DNA levels in ileum organoids or culture medium (0 in *x* axis represents 1 hour postinfection; *n* = 4). (**L**) Infectious viral titers of intracellular virus in ileum organoids (*n* = 3). (**M**) Infectious viral titers of virus secreted into the medium by ileum organoids (*n* = 3). (**N**) Viral DNA levels in rectum organoids and culture medium (*n* = 3). (**O**) Infectious viral titers of intracellular virus in rectum organoids (*n* = 4). (**P**) Infectious viral titers of virus secreted by rectum organoids (*n* = 4). Data are shown as means ± SEM. Values below the gray dashed line indicate undetectable titers.

Gastrointestinal manifestations, particularly proctitis, have been frequently reported during the 2022–2023 global outbreak, which was caused by the clade IIb MPXV strain (*9*, *10*). To investigate whether MPXV can directly infect the intestinal tract, we examined colon tissue from our previously reported fatal mpox case (Fig. 1A) (*19*). In this autopsied patient, numerous lesions were observed in the colon (Fig. 1B). Hematoxylin and eosin (H&E) staining of the descending colon revealed intestinal glands with degenerated goblet cells, characterized by shrunken, eosinophilic cytoplasm and condensed nuclear chromatin, along with exocytosis of inflammatory cells (Fig. 1C). The lamina propria exhibited inflammatory cells with eosinophilic cytoplasm, consistent with Guarnieri-like inclusions, and nuclear alterations. A small venule in the mucosa showed luminal inflammatory cells and an endothelial cell containing a Guarnieri-like inclusion in the cytoplasm (Fig. 1C). Immunohistochemistry (IHC) staining for viral antigens, counterstained with Alcian blue for mucus in goblet cells, revealed numerous infected cells in the lamina propria and goblet cells, with intestinal glands partially or entirely positive for viral antigens (Fig. 1D). Furthermore, colonic epithelium (CDX2 positive) was infected by MPXV, showing cytopathogenic effects including apoptosis and necrosis of epithelial cells (Fig. 1, E to H). IHC staining of the intestinal wall near the peritoneum showed extensive MPXV-positive cells. Notably, MPXV infection was also observed in endothelial and interstitial intestinal cells (Fig. 1I). These findings prompted us to investigate whether human intestinal organoids are permissive to clade IIb MPXV infection using a patient-derived isolate from the 2022 outbreak in the Netherlands.

First, we inoculated the primary organoids isolated from human small intestine (ileum) (Fig. 1J). The quantification of viral DNA by quantitative reverse transcription polymerase chain reaction (qRT-PCR) (table S1) revealed a continuous increase in intracellular viral genome copies from 1 hour to 1 day and up to 7 days postinoculation. The kinetics of extracellular virus secreted into the culture medium showed a similar increasing trend (Fig. 1K). In organoids, plaque assays showed that infectious titers increased from ~3 log_10_ plaque-forming units (PFU)/ml at 1 hour to nearly 6.5 log_10_ PFU/ml at 7 days postinoculation (Fig. 1L). In the culture medium, the infectious titer was undetectable at 1 hour and peaked at nearly 5 log_10_ PFU/ml at 7 days postinoculation (Fig. 1M). Next, we tested organoids isolated from human rectal tissue (Fig. 1J) and consistently observed continuous viral replication within the organoids and viral production into the medium, as measured by both viral DNA levels and infectious titers (Fig. 1, N to P).

**Fig. 2.**
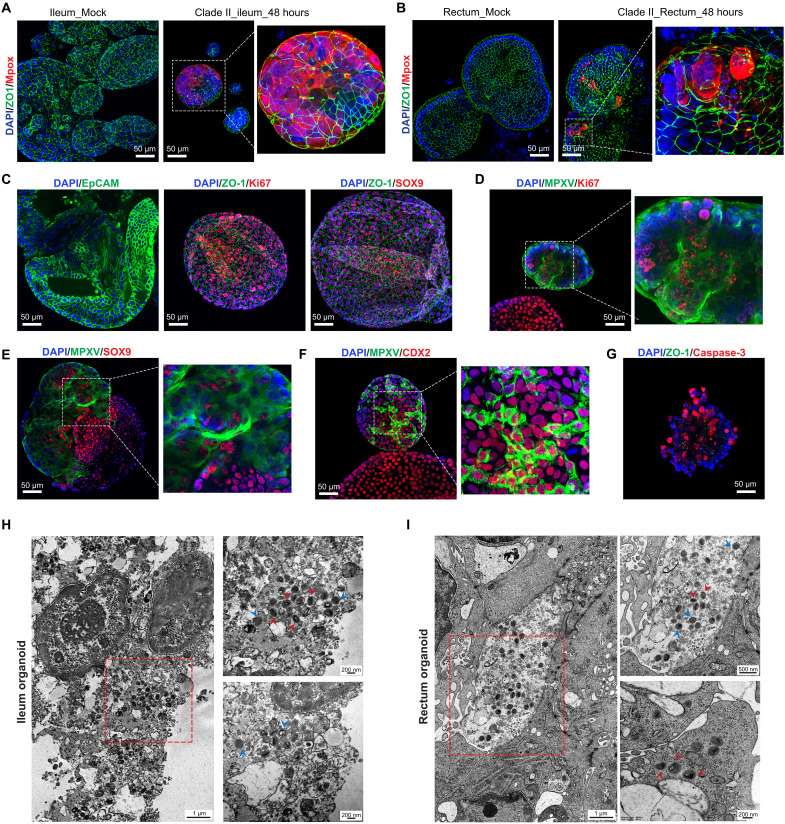
Visualization of MPXV infection in intestinal organoids. (**A**) Immunostaining of MPXV-infected ileum organoids using antibodies against ZO-1 (green), MPXV virions (red), and DAPI (4, 6-diamidino-2-phenylindole) for nuclei (blue). (**B**) Immunostaining of MPXV-infected rectum organoids with the same markers. (**C**) Characterization of intestinal organoids by staining epithelial membrane marker EpCAM, tight junction marker ZO-1, proliferative marker Ki67, and stem cell marker SOX9. (**D** to **F**) Costaining MPXV with Ki67 (D), SOX9 (E), and intestinal epithelium marker CDX2 (F) at 48 hours postinfection. (**G**) Immunostaining of cell death marker cleaved caspase-3 and tight junction marker ZO-1 at 7 days postinfection. (**H** and **I**) Representative TEM visualized MPXV virions in ileum (H) and rectum (I) organoids. Red arrows indicate mature virions, and blue arrows indicate immature virions.

Next, the robust infection was further visualized by immunostaining MPXV virions in both types of organoids (Fig. 2, A and B; fig. S1A; and table S2). Primary intestinal organoids typically exhibit features of both epithelial cells (epithelial cell adhesion molecule, EpCAM^+^) and stem cells (SOX9^+^), along with strong proliferative capacity (Ki67^+^; Fig. 2C). We therefore costained MPXV with these markers and observed that MPXV can infect both proliferating and stem cells (Fig. 2, D and E). We specifically included the intestinal epithelium marker CDX2, and consistent with our clinical findings (Fig. 1, E and F), we found MPXV infection in CDX2-positive organoid cells (Fig. 2F). In addition, infected organoids exhibited marked morphological shrinkage after 7 days of infection, and immunostaining revealed the disruption of tight junctions (ZO-1^+^) and widespread apoptotic cell death (cleaved caspase-3^+^) (Fig. 2G and fig. S2). Transmission electron microscopy (TEM) visualized intracellular MPXV particles, with the majority captured at the immature and mature virion stages in intestinal organoids (Fig. 2, H and I).

### The differential susceptibility of enteric epithelial cell types to MPXV infection

**Fig. 3.**
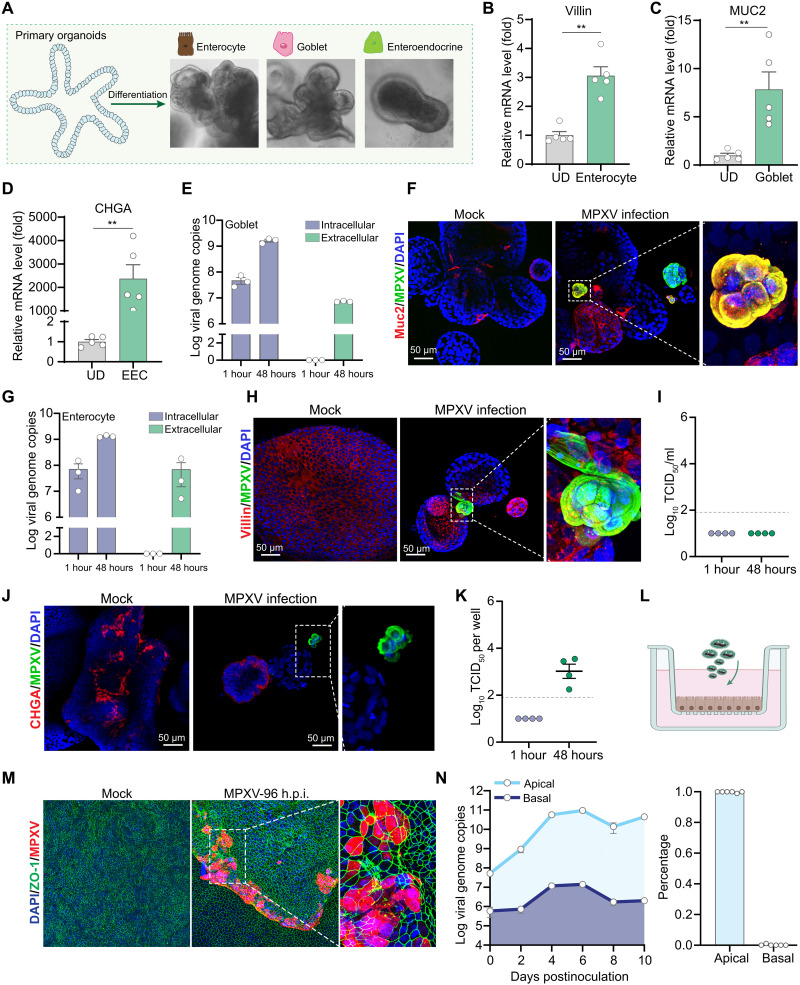
MPXV infection in differentiated intestinal organoids. (**A**) Morphology of intestinal organoids cultured under differentiation protocols to induce enterocyte-, goblet-, and enteroendocrine-cell phenotypes. (**B** to **D**) Gene expression levels of Villin (enterocyte marker), MUC2 (goblet cell marker), and CHGA (enteroendocrine cell marker) upon different differentiation protocols. UD, undifferentiated organoids; EEC, enteroendocrine-differentiated organoids. (**E** and **G**) Quantification of viral DNA levels in goblet-differentiated (E) and enterocyte-differentiated (G) organoids and their respective culture media at 48 hours postinoculation (*n* = 3). (**F**, **H**, and **J**) Immunofluorescence staining of goblet-differentiated (F), enterocyte-differentiated (H), and enteroendocrine-differentiated (J) organoids infected with MPXV for 48 hours. MUC2 (goblet cells, red), villin (enterocytes, red), and CHGA (enteroendocrine cells, red). In enteroendocrine-differentiated organoids, MPXV signals were rarely detected and only in CHGA-negative cells. (**I** and **K**) Quantification of extracellular infectious virus titers in the supernatant (I) and intracellular infectious virus titers in organoids (K) at 48 hours postinfection. (**L**) Schematic diagram of MPXV infection in transwell-cultured organoid cell monolayers. (**M**) Immunostaining of ZO-1 (green), MPXV (red), and nuclei (DAPI, blue) in organoid cell monolayers at 96 hours postinfection. (**N**) Quantification of released viruses from the apical and basolateral compartment of transwell system. Data are presented as means ± SEM. Statistical analysis was performed using the two-tailed Mann-Whitney *U* test. **P < 0.01. Values below the gray line indicate samples with undetectable infectious titers.

To further investigate the infectivity of MPXV across different types of enteric epithelial cells, intestinal organoids were subjected to differentiation culture toward enterocytes, goblet cells, and enteroendocrine cells (Fig. 3, A to D, and fig. S3A). These differentiated organoids were subsequently inoculated with clade IIb MPXV particles. The qRT-PCR quantification of viral DNA indicated that organoids differentiated into goblet cells and enterocytes readily supported MPXV replication and production (Fig. 3, E and G). The immunostaining of MPXV virions further confirmed infection in goblet cell– and enterocyte-differentiated organoids (Fig. 3, F and H). In contrast, infectious virus titers were undetectable in the supernatant of enteroendocrine-differentiated organoids at both 1 and 48 hours postinoculation (h.p.i.) (Fig. 3I). Moreover, MPXV fluorescence signals were not detected in CHGA-positive cells (fig. S3B), although low-frequency MPXV positivity was observed in CHGA-negative cells (Fig. 3J). This aligns with the low infectious titers detected at 48 h.p.i. in organoids (Fig. 3K). However, no increase in viral DNA levels was observed in these organoids (fig. S3C), suggesting masking by the input viral inoculum and minimal viral replication.

In addition, we used ileal organoid-derived cells to grow epithelial monolayers on a permeable transwell support. Upon viral inoculation from the apical (luminal) compartment (Fig. 3L), robust viral infection was observed by immunostaining at 48 h.p.i. (Fig. 3M). qRT-PCR quantification showed viral secretion into both the apical (up to 11 log_10_ copies) and basolateral (up to 7 log_10_ copies) compartments, although more than 99% of the total viral production occurred in the apical compartment (Fig. 3N).

### Transcriptomic analyses reveal active MPXV–host interactions

**Fig. 4.**
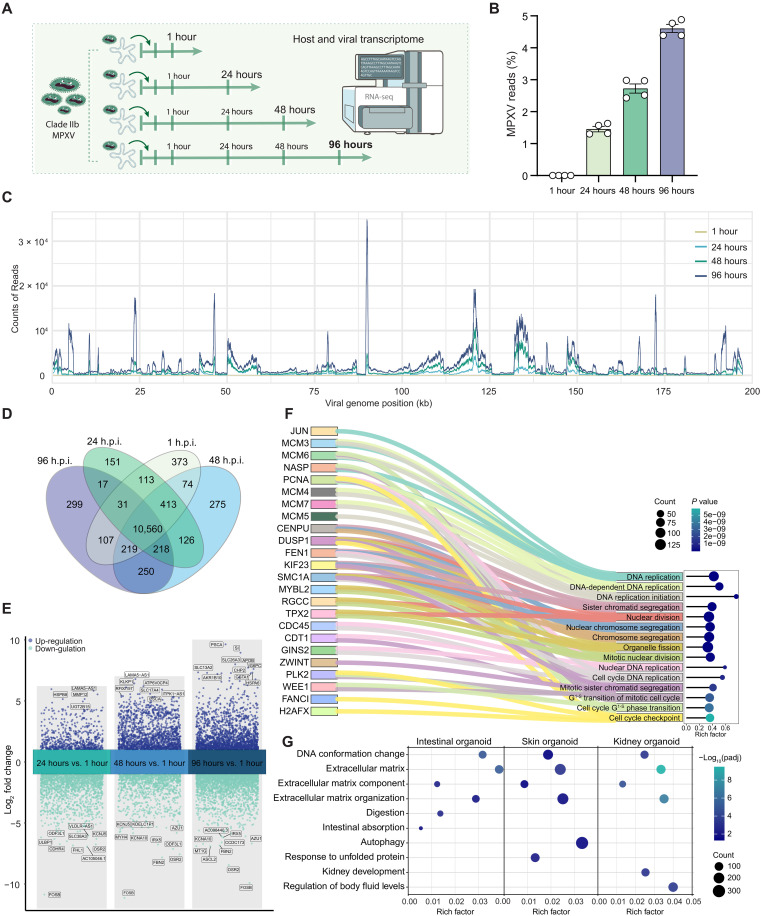
Mapping host and virus transcriptomes. (**A**) Schematic representation of bulk RNA sequencing groups. (**B**) Percentage of mapped MPXV transcripts across different organoid groups. (**C**) Distribution of MPXV transcripts mapped to specific regions of the viral genome. (**D**) Venn diagram showing overlap of differentially expressed genes in MPXV-infected organoids at various time points. (**E**) Volcano plot analysis of differentially expressed genes at 24, 48, and 96 hours postinfection compared to 1 hour postinoculation (h.p.i.). (**F**) Gene Ontology (GO) enrichment analysis of differentially expressed genes in human intestinal organoids infected with MPXV (96 h.p.i versus 1 h.p.i.). (**G**) Comparison of enriched signaling cascades among MPXV-infected intestinal, skin, and kidney organoids.

As a large DNA virus, MPXV transcribes hundreds of viral genes during infection (*20*), following a cascade temporal expression pattern (*21*). We performed transcriptomic analysis on intestinal organoids infected with clade IIb MPXV (Fig. 4A) and notably observed a gradual increase in MPXV transcript levels over 96 h.p.i. (Fig. 4B). In-depth analysis revealed numerous abundantly expressed transcripts mapped to various regions of the MPXV reference genome, showing distinct temporal expression patterns (Fig. 4C). Notably, a cascade of transcripts encoding key viral proteins—including OPG015 (ankyrin repeat protein), OPG039 (ankyrin-like protein), and OPG200 (Bcl-2–like protein)—markedly increased after 48 h.p.i. (fig. S4).

The comparison of differentially expressed host genes across time points postinoculation revealed that hundreds of genes were uniquely expressed at each time point, while more than 10,000 genes were consistently expressed across all time points (Fig. 4D). Volcano plot analysis showed robust host responses to MPXV infection at 24, 48, and 96 h.p.i. compared to 1 h.p.i., with thousands of genes significantly up- or down-regulated (Fig. 4E). Intriguingly, several prominently regulated genes—such as *OSR2*, *FOSB*, and *PSCA*—were consistently dysregulated from 24 to 96 h.p.i. Gene Ontology (GO) analysis revealed that at 48 h.p.i., pathways related to ion transport and immune response were significantly enriched (fig. S5A), whereas at 96 h.p.i., many significantly regulated pathways were associated with DNA replication compared to 1 h.p.i. (fig. S5B). Sankey diagram further visualized the most significantly regulated genes within enriched GO biological processes (Fig. 4F), underscoring that MPXV infection at 96 hours profoundly affects host DNA synthesis. In addition, transcriptional signatures related to unfolded protein responses and heat stress responses were enriched in infected organoids at 96 h.p.i. compared to uninfected controls (fig. S6, A to C). We next compared transcriptional alterations in intestinal organoids with our previous studies on MPXV-infected skin and kidney organoids (*22*, *23*). GO analysis revealed both shared enriched signaling pathways and organ-specific biological processes (Fig. 4G). Notably, more than 200 genes were commonly up-regulated or down-regulated across the three organoid types (fig. S7 and tables S3 and S4), suggesting shared core host response pathways to MPXV infection. Overall, these findings demonstrate that MPXV, with its large DNA genome encoding numerous viral proteins, elicits specific and dynamic host responses in intestinal organoids throughout the course of infection.

### Drug screening in intestinal organoids identified leading candidates against clade IIb MPXV

**Fig. 5.**
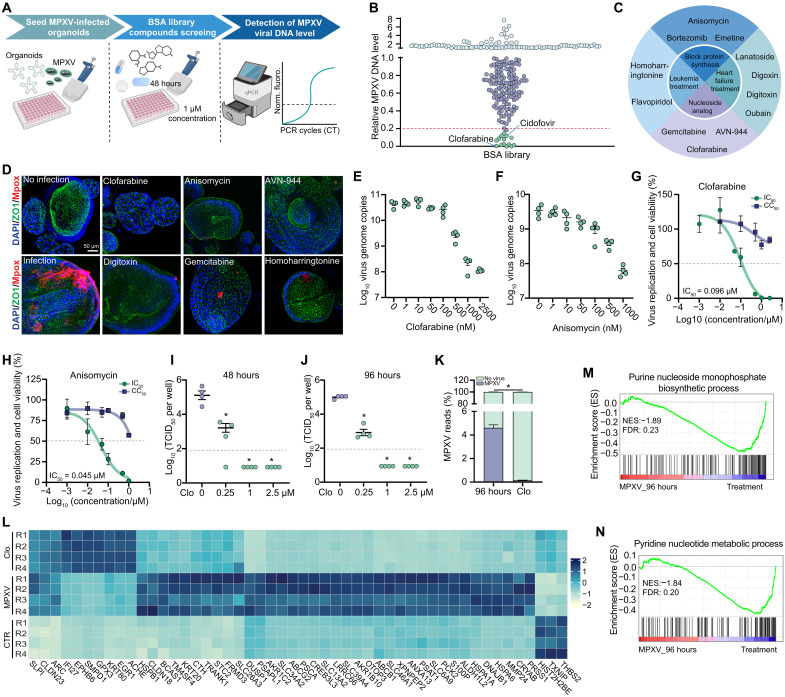
Antiviral drug discovery and validation in MPXV-infected intestinal organoids. (**A**) Schematic diagram of BSA library screening in MPXV-infected intestinal organoids. (**B**) Quantification of MPXV DNA levels following treatment with 240 compounds from the BSA library for 48 hours. (**C**) Summary of the 12 identified potent MPXV inhibitors. (**D**) Immunostaining of MPXV virions in intestinal organoids at 48 hours postinfection showing the inhibitory effects of six prioritized compounds. (**E** and **F**) Inhibitory activity of clofarabine (E) and anisomycin (F) against MPXV DNA in intestinal organoids treated for 48 hours. (**G** and **H**) Half maximum inhibitory concentration (IC_50_) and half maximum cytotoxic concentration (CC_50_) of clofarabine (G) and anisomycin (H) in MPXV-infected intestinal organoids for 48 hours treatment. (**I** and **J**) Inhibitory efficacy of clofarabine (Clo) on intracellular MPXV infectious titers in intestinal organoids after 48 hours (I) and 96 hours (J) of treatment. (**K**) Percentages of mapped MPXV transcripts in organoids treated with 1 μM clofarabine or untreated after 96 hours of infection (*n* = 4, *P < 0.05 by χ^2^ test). (**L**) Top 50 significantly regulated genes following MPXV infection for 96 hours. (**M** and **N**) Gene set enrichment analysis showing the regulation of the purine nucleoside monophosphate biosynthetic process (M) and pyridine nucleotide metabolic process (N) in MPXV-infected organoids compared to MPXV infection with clofarabine treatment. Data are presented as means ± SEM. Statistical analysis was performed using the two-tailed Mann-Whitney *U* test, unless specified. *P < 0.05. Values below the gray line indicate samples with undetectable infectious titers.

Effective antiviral treatments for MPXV infection are urgently needed (*24*). A unique advantage of primary intestinal organoids is their capacity for rapid and large-scale expansion (*25*), enabling us to screen a library of 240 known safe-in-human broad-spectrum antiviral (BSA) agents in clade IIb MPXV-infected intestinal organoids (Fig. 5A). To minimize nonspecific effects, we used a low concentration (1 μM), with cidofovir—a known poxvirus inhibitor—included as a control. After 48 hours of treatment, 12 compounds were identified with more than 80% inhibition of intracellular MPXV genomic DNA (Fig. 5B), showing potency equivalent to or greater than cidofovir. These inhibitors were categorized into four groups based on their original clinical applications: heart failure treatment, leukemia treatment, protein synthesis blockers, and nucleoside analogs (Fig. 5C). qRT-PCR quantification demonstrated that six selected compounds potently and dose dependently inhibited intracellular MPXV DNA levels (fig. S8, A to F). Immunostaining of MPXV virions further confirmed the strong inhibitory effects of these agents (Fig. 5D), and their anti-MPXV activity was validated in human rectal organoids (fig. S8G).

Notably, clofarabine and anisomycin emerged as the most potent inhibitors of MPXV infection, reducing MPXV DNA copies by ~2.5 log_10_ and 1.8 log_10_, respectively (Fig. 5, E and F). The estimated half-maximal inhibitory concentration (IC_50_) of clofarabine and anisomycin was 0.096 and 0.045 μM, respectively, with no major cytotoxicity observed at the tested concentrations (Fig. 5, G and H, and fig. S8, H and I). In clinical settings, the antiviral treatment for patients with mpox is often delayed (*26*). To simulate this, we initiated a 48-hour treatment with 1 μM clofarabine or anisomycin at 3 days postinfection. The quantification of intracellular MPXV DNA showed a nearly 2 log_10_ reduction in viral DNA copies (fig. S9A), and plaque assays demonstrated significant inhibition of infectious virus production in the culture medium (fig. S9B). We also modeled a longer-term treatment scenario, initiating therapy immediately after viral inoculation and extending it for 7 days. This resulted in significant inhibition of both intracellular viral replication and extracellular virus production (fig. S9, C and D).

### Validation of clofarabine as a potent inhibitor against clade IIb MPXV in intestinal and skin organoids

Next, we prioritized clofarabine for further validation considering its favorable pharmacological profiles and its prior FDA approval for the treatment of acute lymphoblastic leukemia (*27*). We quantified infectious virus titers in MPXV-infected intestinal organoids following treatment with different concentrations of clofarabine for 48 or 96 hours. Median tissue culture infectious dose (TCID_50_) assays showed that 0.25 μM clofarabine reduced viral titers by nearly two log_10_, while viral titers became undetectable at 1 and 2.5 μM (Fig. 5, I and J). To benchmark its antiviral potency, we also tested tecovirimat and brincidofovir—two antivirals approved for smallpox and used compassionately for mpox, although with inconclusive clinical efficacy. In our model, 1 μM tecovirimat led to only a modest reduction in intracellular viral titers in intestinal organoids, whereas brincidofovir showed strong inhibitory effects (fig. S9E).

Subsequently, genome-wide transcriptomic analysis was performed on intestinal organoids infected with MPXV for 96 hours, with or without clofarabine treatment. We observed a marked reduction in MPXV transcripts in clofarabine-treated intestinal organoids compared to untreated controls at 96 hours (4.61% versus 0.14%; 97% inhibition) (Fig. 5K). Differential gene expression analysis indicated that clofarabine treatment largely prevented the extensive host transcriptome rewiring induced by MPXV infection (Fig. 5L and fig. S10, A to C). The correlation of significantly regulated genes with enriched KEGG pathways revealed prominent activation of several key signaling cascades, including calcium and mitogen-activated protein kinase (MAPK) signaling pathways (fig. S10D). Consistent with a previous study showing activation of the STING –nuclear factor κB (NF-κB) pathway in cancer cells treated with clofarabine (*28*), we found that the NF-κB signaling pathway was markedly activated in clofarabine-treated organoids (fig. S11). Gene set enrichment analysis revealed that transcriptional signatures associated with nucleoside biosynthetic processes were largely suppressed by clofarabine treatment (Fig. 5, M and N, and fig. S6, D to G). Collectively, the impact on host transcriptomics is likely attributable to a combination of direct effects from clofarabine and indirect effects mediated through inhibition of MPXV infection.

**Fig. 6.**
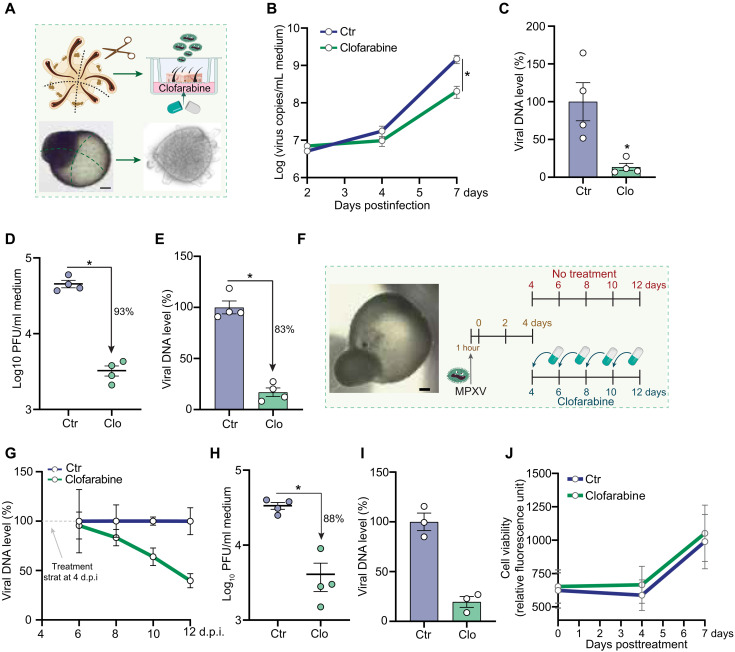
Validation of the anti-MPXV activity of clofarabine in human skin organoids. (**A**) Schematic illustration of ALI-cultured skin organoids. (**B**) Quantification of MPXV DNA level in the culture medium of ALI-cultured skin organoids at 2, 4, and 7 days postinfection (*n* = 4). (**C**) Relative viral genome DNA levels at 7 days postinfection (*n* = 4). (**D**) Quantification of MPXV infectious titers in the culture medium at 7 days postinfection (*n* = 4). (**E**) Quantification of MPXV DNA levels in skin organoids at 7 days postinfection (*n* = 4). (**F**) Cell viability of skin organoids with or without clofarabine treatment (1 μM). (**G**) Schematic representation of delayed clofarabine treatment in skin organoids. (**H**) Quantification of MPXV DNA level in culture medium at 6, 8, 10, and 12 days postinoculation. Clofarabine (1 μM) was administered at 4 days postinoculation (*n* = 4). (**I**) Quantification of infectious titers in the culture medium of skin organoids at 12 days postinoculation (*n* = 4). (**J**) Quantification of MPXV DNA levels in skin organoids at 12 days postinoculation (*n* = 3). h.p.i., hour postinoculation; d.p.i., day postinoculation. Data are presented as means ± SEM. Statistical analysis was performed using the two-tailed Mann-Whitney *U* test. *P < 0.05.

Recently, human induced pluripotent stem cell–derived skin organoids have been demonstrated to serve as robust models for MPXV infection and therapeutic testing (*6*). Here, we further validated the antiviral activity of clofarabine in air-liquid interface (ALI)–cultured skin organoids (Fig. 6A). We first assessed the antiviral effect of immediate treatments over 7 days following viral inoculation. This resulted in a significant inhibition of virus production in the culture medium at day 7 (Fig. 6, B and C). Correspondingly, infectious titers of the produced virus were potently inhibited, with 93% reduction shown by plaque assay (Fig. 6D). Consistently, intracellular viral replication was strongly suppressed, as demonstrated by quantification of viral DNA in organoids after 7 days of treatment (Fig. 6E).

Next, we performed a delayed treatment in cystic skin organoids, initiating clofarabine (1 μM) administration 4 days postinfection (Fig. 6F). The quantification of MPXV DNA levels in the culture medium showed persistent inhibition of virus production from day 6 to 12 postinfection (Fig. 6G). On day 12, treatment resulted in an 88% reduction of infectious virus titers (Fig. 6H). Likewise, intracellular virus replication was potently inhibited, as shown by viral DNA quantification in skin organoids on day 12 postinfection (Fig. 6I). Cell viability assay confirmed that 1 μM clofarabine treatment caused no detectable cytotoxicity in skin organoids (Fig. 6J).

### Clofarabine inhibits clade Ia and Ib MPXV infections in intestinal organoids

**Fig. 7.**
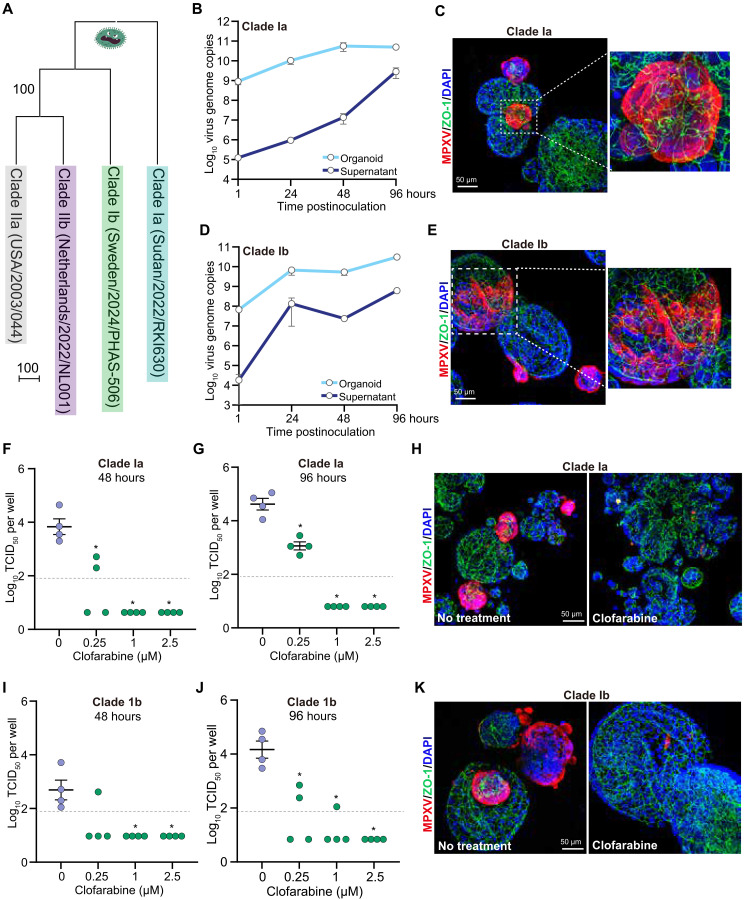
Modeling clade Ia and Ib MPXV infections and validating the antiviral effects of clofarabine in intestinal organoids. (**A**) Phylogenetic tree of clade I and II MPXV strains. (**B**) Quantification of viral DNA levels in intestinal organoids and culture medium at 1, 24, 48, and 96 hours postinoculation with clade Ia MPXV strain (*n* = 4). (**C**) Representative immunostaining of clade Ia MPXV-infected intestinal organoids using antibodies against MPXV virions (red), ZO-1 (red), and DAPI for nuclei (blue). (**D**) Quantification of viral DNA levels in intestinal organoids and culture medium at 1, 24, 48, and 96 hours postinfection with clade Ib MPXV strain (*n* = 4 to 5). (**E**) Representative immunostaining of clade Ib MPXV-infected intestinal organoids using antibodies against MPXV virions (red), ZO-1 (red), and DAPI (blue). (**F** and **G**) Quantification of the intracellular infectious virus titers in intestinal organoids infected with clade Ia MPXV at 48 (F) and 96 (G) hours postinfection (*n* = 4). (**H**) Representative immunostaining of clade Ia MPXV-infected intestinal organoids treated or untreated with clofarabine at 48 hours postinfection using antibodies against MPXV virions (red), ZO-1 (red), and DAPI dye (blue). (**I** and **J**) Quantification of the intracellular infectious virus titers in intestinal organoids infected with clade Ib MPXV at 48 (I) and 96 (J) hours postinoculation (*n* = 4). (**K**) Representative immunostaining of clade Ib MPXV-infected intestinal organoids treated or untreated with clofarabine at 48 hours postinoculation using antibodies against MPXV virions (red), ZO-1 (red), and DAPI dye (blue). Data are presented as means ± SEM. Statistical analysis was performed using the two-tailed Mann-Whitney *U* test. *P < 0.05. Values below the gray line indicate samples with undetectable infectious titers.

Currently, mpox outbreaks caused by the clade Ia and Ib strains are devastating in Central and East Africa (*3*). We therefore aimed to determine whether intestinal organoids are also susceptible to their infection and whether clofarabine can also inhibit infection of these strains. To this end, we inoculated intestinal organoids with patient-derived clade Ia and Ib MPXV isolates (Fig. 7A). For the clade Ia isolate, we observed a continuous increase in both intracellular and extracellular viral genome copies from 1 to 96 h.p.i. (Fig. 7B). Immunofluorescence staining further confirmed robust MPXV infection at 48 h.p.i. (Fig. 7C). Similarly, intestinal organoids effectively supported replication and production of clade Ib MPXV, as demonstrated by qPCR quantification of viral genomic DNA and immunostaining of MPXV virions (Fig. 7, D and E).

Last, we treated intestinal organoids infected with clade Ia or Ib MPXV using clofarabine. For the clade Ia strain, treatment with 1 μM clofarabine for 48 hours inhibited 98% of intracellular viral DNA replication and reduced infectious virus production in the culture medium by 76% (fig. S12, A and B). TCID_50_ assays further demonstrated potent antiviral efficacy, with intracellular viral titers becoming undetectable at clofarabine concentrations of 1 and 2.5 μM after 48 or 96 hours of treatment (Fig. 7, F and G). Immunostaining confirmed this strong inhibitory effect against clade Ia MPXV (Fig. 7H). Consistently, clofarabine also inhibited clade Ib MPXV replication and extracellular virus production, as demonstrated by the quantification of viral genomic DNA, infectious virus titers, and immunostaining of virions (Fig. 7, I to K, and fig. S12, C and D). Collectively, these results show that clofarabine is effective against both clade Ia and Ib MPXV strains.

## DISCUSSION

In general, common symptoms of mpox—such as skin rash or mucosal lesions—typically resolve within a few weeks. However, some patients may develop disseminated disease, leading to severe complications and even death. The histopathological evaluation of autopsy and biopsy tissues from fatal mpox cases with systemic manifestations has shown the presence of MPXV antigen and viral DNA in a wide range of tissues, including skin, ocular, oropharyngeal, and mucosal digestive tract tissues (*29*). Gastrointestinal symptoms, particularly proctitis, were frequently reported during the 2022–2023 global outbreak caused by the clade IIb strain (*9*). Historical reports, including those predating the recent outbreak, have documented gastrointestinal symptoms such as diarrhea among mpox patients infected with clade I MPXV, presumably clade 1a, although sequence confirmation was often unavailable (*9*). Recent data from the ongoing mpox outbreak in the DRC (Democratic Republic of the Congo), attributed to clade 1b MPXV, have also reported gastrointestinal symptoms, such as reporting diarrhea in 13% of confirmed cases (*30*).

In our autopsied mpox patient (*19*), we observed robust MPXV infection and extensive lesions in the colon. Consistent with these clinical observations, we demonstrated that primary organoids cultured from human small intestine and rectum are highly susceptible to MPXV infection. Furthermore, our model captured MPXV-induced cell death, aligning with the severe tissue damage observed in the patient’s intestine. These organoids, derived from tissue stem cells and cultured in a three-dimensional (3D) structure, are capable of recapitulating the architecture, cellular composition, diversity, organization, and functionality of the original intestinal tissue (*31*). We further differentiated intestinal organoids into three types of enteric cells. Enterocytes and goblet cells support robust MPXV replication, whereas the level of infection in enteroendocrine cells appears substantially lower. A recent study using human induced pluripotent stem cell–derived colon organoids largely failed to model MPXV infection (*32*). Although the underlying reason remains unclear, we speculate that their model may lack the cell types permissive to MPXV replication. When our organoids were grown as epithelial monolayers on a transwell system in 2D, MPXV was predominantly secreted into the apical compartment, although basolateral secretion also occurred. This may reflect gastrointestinal shedding of MPXV, as the virus has been detected in stool samples from a proportion of patients with mpox (*33*).

We demonstrated the utility of intestinal organoids for assessing antiviral drugs. Tecovirimat and brincidofovir are approved antiviral drugs for treating smallpox and have been widely prescribed under compassionate use for mpox (*17*). In cell lines, tecovirimat shows extremely potent anti-MPXV activity, with reported IC_50_ values ranging from 4 to 20 nmol, which is stronger than brincidofovir (9 to 152 nmol) (*34*). Our findings in intestinal organoids confirmed their inhibitory effects as observed in MPXV-infected cell lines. However, in our organoid model, tecovirimat at 1 μM achieved only partial to moderate reduction in infectious virus titers, whereas the same concentration of brincidofovir resulted in near-complete inhibition. These observations in organoids may help explain the limited clinical efficacy of tecovirimat reported in two large-scale clinical trials (*15*, *17*). Nevertheless, the clinical scenario is likely more complex. By disrupting the major envelope-wrapping protein VP37 of orthopoxviruses, tecovirimat only prevents infectious virus production but does not affect other steps of the viral life cycle (*35*). Thus, tecovirimat may not be potent enough to completely eradicate the virus in infected patients.

A unique advantage of primary organoids is their capacity for rapid expansion, enabling small- to medium-scale drug screening (*36*). In this study, we focused on BSAs, which can inhibit multiple viruses from the same or different families by targeting conserved viral components or shared host cellular pathways (*37*). The broad nature of their antiviral activity increases the likelihood of effectiveness against newly emerging viruses, making BSAs an attractive strategy for responding to epidemics such as the mpox outbreaks (*38*). Through screening our BSA library, we identified 12 drug candidates that were comparable to or more potent than the control cidofovir in inhibiting MPXV replication. We prioritized safe-in-human BSAs, which are already used clinically or that have passed phase 1 trials, and therefore, these candidates can be rapidly advanced into clinical testing for this new indication (*39*).

We identified clofarabine, an FDA-approved drug for treating relapsed or refractory acute lymphoblastic leukemia in children (*27*), as a potent inhibitor of MPXV. A previous study reported an EC_50_ value of 0.92 μM for clofarabine against the modified vaccinia Ankara strain but noted cytotoxic effects in their model (*40*). In contrast, we observed that clofarabine at 1 μM exerts minimal or no toxicity in intestinal and skin organoids. This discrepancy may be explained by the high sensitivity of their cancer cell line models to this anticancer drug (*40*). In line with our observations, prior pharmacokinetic and pharmacodynamic studies reported a median plasma clofarabine concentration of 1.5 μM (range: 0.42 to 3.2 μM) and a median intracellular concentration of 19 μM (range: 3 to 52 μM) (*41*). In this study, we determined the IC_50_ value of clofarabine for MPXV inhibition to be as low as around 0.1 μM, and extensive validation using 1 μM showed complete inhibition of infectious titers in treated organoids. These effective concentrations are clinically relevant and achievable in treated patients (*41*, *42*). We further validated clofarabine’s anti-MPXV activity in human skin organoids (*23*). However, its antiviral potency appeared greater in intestinal organoids compared to skin organoids, possibly due to lower drug bioavailability in the skin organoid model (*23*). Currently, clofarabine is administered via intravenous infusion, which generally ensures high systemic bioavailability. Nonetheless, topical formulations may be considered to enhance skin bioavailability for treating mpox-related skin lesions (*43*).

The epidemiological and clinical features of mpox vary substantially depending on the viral (sub-)clades and the specific context. The 2022–2023 global outbreak of clade IIb primarily affected men who have sex with men through sexual network (*9*). Historically, clade I MPXV was considered more pathogenic (*44*), with transmission unrelated to sexual activity. However, the ongoing outbreak of clade I in DRC has documented evidence of sexual transmission (*45*). While anyone can be infected with MPXV, vulnerable populations such as children, pregnant women, and immunosuppressed individuals (e.g. those infected with HIV) are at higher risk of developing severe complications (*46*). Currently, clade Ia and Ib strains are cocirculating in Africa (*3*), with the emerging clade Ib variant harboring novel mutations (*4*). We have demonstrated that human primary intestinal organoids can model the infections of clade IIb, Ia, and Ib strains and that clofarabine is highly effective against all three tested strains.

As a purine nucleoside analog, clofarabine has been shown to inhibit HIV replication through both host- and virus-targeted mechanisms, by suppressing cellular nucleotide synthesis and directly inhibiting the DNA polymerase activity of HIV reverse transcriptase (*47*). Although the anti-MPXV mechanisms of clofarabine remain to be elucidated, they are likely distinct from the mode of action of tecovirimat, which directly targets the viral protein VP37 to prevent infectious virus production (*13*). Given their complementary mechanisms, it would be valuable to assess the combination of clofarabine and tecovirimat for potential synergistic antiviral activity and to reduce the risk of drug resistance (*48*). Notably, clofarabine has known side effects, including hepatotoxicity, nephrotoxicity, and bone marrow suppression (*49*), which caution against its immediate clinical translation for treating mpox. However, our findings support future research to evaluate the potential for repurposing clofarabine in different therapeutic contexts for mpox, considering treatment duration, dosage, and route of administration (e.g., reformulation as topical creams for skin lesions). Clofarabine may be particularly attractive for mpox patients with acute lymphoblastic leukemia.

There are some limitations in this study. First, we did not compare our organoids with conventional models such as cell lines. Different models can serve complementary roles depending on the scientific question, and our organoid system adds an important layer of complexity for evaluating host-pathogen interactions and drug responses. Second, our models support robust infections by different MPXV subclades, but they do not yet fully recapitulate their (differential) disease manifestations, partly due to the absence of immune cells within the organoid system. Third, while clofarabine has shown promising anti-MPXV activity, further evaluation is needed before advancing to clinical testing for mpox treatment. Its antiviral mechanisms should also be explored in future research. Fourth, our organoid models primarily represent acute MPXV infection and short-term antiviral treatment. However, prolonged mpox may occur in specific cases (e.g., individuals with advanced HIV) (*50*), and these patients may require extended treatment. Future research should therefore extend to developing experimental models that mimic persistent MPXV infection, assess long-term treatment strategies, and monitor potential emergence of drug resistance. Last, we did not test clade IIa. Although this subclade has historically been limited to a few West African countries, with low human-to-human transmission and minimal impact on recent mpox outbreaks (*51*), cases were reported in Côte d’Ivoire, Guinea, and Liberia in 2024, marking the first evidence of sustained community transmission (*52*). This warrants increased attention in future studies.

In summary, this study successfully modeled infections by clade Ia, Ib, and IIb MPXV isolates in human intestinal organoids and demonstrated the potent antiviral activity of clofarabine across these three subclades. Our innovative experimental model and drug discovery pipeline have significant implications for addressing the current mpox global health emergency and enhancing preparedness for future poxvirus epidemics.

## MATERIALS AND METHODS

### Intestinal organoids culture

Human primary intestinal organoids were isolated and cultured as we previously described (*25*, *53*). The use of human intestinal tissue for research purpose including culturing into organoids was approved by the Medical Ethical Council of the Erasmus MC, and informed consent was given (MEC-2021-0432 and MEC-2023-0629). These organoids were cultured in organoid expansion medium (OEM) based on advanced Dulbecco’s modified Eagle’s medium (DMEM)/F12 (Invitrogen) supplemented with 1% penicillin/streptomycin (Life Technologies), 10 mM Hepes, 1x GlutaMAX, 1 mM N2, 1 mM B27 (all from Invitrogen), 1 μM *N*-acetylcysteine (Sigma-Aldrich), and the following growth factors: mouse epidermal growth factor (50 ng/liter), 50% Wnt3a-conditioned medium (WCM) and 10% noggin-conditioned medium, 20% Rspo1-conditioned medium, 10 μM nicotinamide (Sigma-Aldrich), 10 nM gastrin (Sigma-Aldrich), 500 nM A83-01 (Tocris), and 10 μM SB202190 (Sigma-Aldrich). The medium was refreshed every 2 to 3 days, and organoids were passaged 1:3 every 5 to 7 days.

### Differentiation of organoids

Organoids were further differentiated toward different cell types by using the respective differentiation medium. Briefly, organoids were cultured in OEM (without WCM) supplemented with 2 μM IWP-2 (Sigma-Aldrich) for enterocytes differentiation; OEM (without WCM) supplemented with 2 μM IWP-2 and 10 μM DAPT (MedChemExpress) for goblet cells differentiation; and OEM (without WCM) supplemented with 2 μM IWP-2, 10 μM DAPT, and 5 μM Gefitinib (Sigma-Aldrich) for enteroendocrine cell differentiation. The differentiation medium was refreshed every 2 to 3 days, and expected cell types were achieved after 5 days differentiation culture.

### Virus inoculation

Intestinal organoids were mechanically fragmented and then exposed to MPXV viral particles for 1 hour at 37°C. Every 1000 organoids were exposed to ~10^4^ PFU viruses. To increase the infection efficacy, organoids and virus mixture were resuspended every 20 min throughout the inoculation period. Subsequently, fragmented organoids underwent centrifugation at 300*g* for 5 min at 4°C, and the supernatant was discarded. Then, organoids were thoroughly washed three times with advanced DMEM/F12 to remove residual viruses. After infection, the organoids were embedded in Matrigel and cultured in OEM.

### Organoid cells cultured in transwell system

Intestinal organoids were digested into single cells by TrypLE Express, and ~2.4 × 10^4^ cells were then seeded in each transwell insert (precoated with 10-fold diluted Matrigel), with 200 and 450 μl of OEM supplemented in the apical and basolateral compartment respectively. After cells growing into full confluence, MPXV viral particles were inoculated from apical side of the insert.

### AlamarBlue assay

Culture supernatant was discarded, and the organoids were incubated with a 1:20 dilution of AlamarBlue regent (Invitrogen, DAL1100) in culture medium for 2 hours at 37°C. Subsequently, 100-μl medium was collected to assess cell metabolic activity, with each sample being measured in duplicate. Absorbance measurements were obtained using a fluorescence plate reader (CytoFluor Series 4000, PerSeptive Borganoidsystems) at an excitation wavelength of 530/25 nm and an emission wavelength of 590/35 nm.

### DNA extraction and qPCR detection

Total DNA was purified from infected organoids or culture medium using the Macherey-Nagel NucleoSpin DNA Kit (Bioke, Netherlands) and quantified by NanoDrop ND-1000 (Wilmington, USA). Viral DNA levels were quantified by SYBR Green-based qRT-PCR (Applied Biosystems SYBR Green PCR Master Mix, Thermo Fisher Scientific Life Sciences) with the StepOnePlus System (Thermo Fisher Scientific Life Sciences). MPXV viral DNA was calculated by previously generated formula “*y* = −0.3095*x* + 15.387.” The primers used in this study were provided in table S1.

### Plaque assay and TCID_50_ assay

Organoids were collected and stored in 1-ml serum-free advanced DMEM/F12 medium (with 1x GlutaMAX, 1 M Hepes, and 1% penicillin/streptomycin) and centrifuged after three cycles of freezing and thawing to collect clear cell lysates as intracellular viruses. Cleared supernatants from culture medium were used as extracellular viruses. For plaque assay, confluent Vero cells in 12-well plates were washed once with phosphate-buffered saline (PBS) before virus inoculation. Next, Vero cells were overlaid with 1.2% avicel in serum-free advanced DMEM/F12 medium (with 1x GlutaMAX, 1 M Hepes, and 1% penicillin/streptomycin) containing 10-fold serial dilutions of samples. Plates were incubated 4 days at 37°C and then fixed by 4% paraformaldehyde (PFA) and stained with 0.1% crystal violet. Plaques were quantified as PFU/ml. For TCID_50_ assay, 10-fold serial dilutions of MPXV viruses were inoculated onto Vero cells that grown in 96-well culture plates at 2000 cells per well. The plate was incubated 4 to 5 days at 37°C, and cells were examined under a light microscope for cytopathic effect. The TCID_50_ value was calculated by using the Reed-Muench method (*54*). Data were presented as the common logarithm, and the samples with undetectable plaque were arbitrarily denoted with a value “1.”

### Immunofluorescence staining and confocal imaging

Organoids were fixed with 4% PFA for 15 min. Then, the samples were gently rinsed three times with PBS, followed by permeabilizing with PBS containing 0.2% (v/v) Triton X-100 for 10 min. Next, samples were twice rinsed with PBS for 5 min, followed by incubation with blocking solution (5% donkey serum, 1% bovine serum albumin, and 0.2% Triton X-100 in PBS) at room temperature for 1 hour. Primary antibodies diluted in blocking solution were subsequently incubated with samples at 4°C overnight. Samples were then washed three times for 5 min each in PBS before 1 hour incubation with 1:1000 dilutions of the secondary antibodies. Nuclei were stained with DAPI (4, 6-diamidino-2-phenylindole; Invitrogen). Last, stained samples were visualized using a Leica SP5 confocal microscope to analyze the stained cellular structures. Antibodies used in this study were listed in the table S2.

### Genome-wide RNA sequencing and data analysis

Intestinal organoids cultured from human ileum tissue were infected with MPXV clade IIb (NL001 strain) viral particles as above description. Infected organoids were harvested at 1, 24, 48, and 96 hours postinfection. Infected organoids with 1 μM clofarabine treatment starting from 1 to 96 hours were set up as a treatment group. In parallel, uninfected organoids were cultured under same conditions for 96 hours as negative controls. Total RNA was isolated using the MachereyNagel NucleoSpin RNA II Kit (Bioke, Netherlands) and quantified with the Bioanalyzer RNA 6000 Picochip. Afterward, RNA sequencing was conducted by Novogene using a paired-end 150–base pair (PE 150) sequencing strategy.

### Statistics

Statistical analysis was performed using GraphPad Prism8 statistics software (GraphPad, San Diego, USA). All data are presented as means ± SEM. Comparison between two groups was analyzed by Mann-Whitney *U* test. Asterisks indicated the degree of significant differences compared with the controls (*P < 0.05 and **P < 0.01).
