# Supplementary material for: Primary human intestinal organoids model enteric infection of monkeypox virus and enable scalable drug discovery
Source: Sci Adv. 2026 Mar 27;12(13):eaea8280. doi: 10.1126/sciadv.aea8280 (PMC13025031; doi:10.1126/sciadv.aea8280)
Supplement: Supplementary file 1 — Figs. S1 to S12 Tables S1 to S4 [file sciadv.aea8280_sm.pdf]

Science Advances
AAAS

Supplementary Materials for

Primary human intestinal organoids model enteric infection of monkeypox virus and enable scalable drug discovery

Pengfei Li *et al.*

Corresponding author: Pengfei Li, p.li@erasmusmc.nl; Marcel J. C. Bijvelds, m.bijvelds@erasmusmc.nl; Qiuwei Pan, q.pan@erasmusmc.nl

Sci. Adv. **12**, eaea8280 (2026)
DOI: 10.1126/sciadv.aea8280

This PDF file includes:

Figs. S1 to S12
Tables S1 to S4

Figure S1

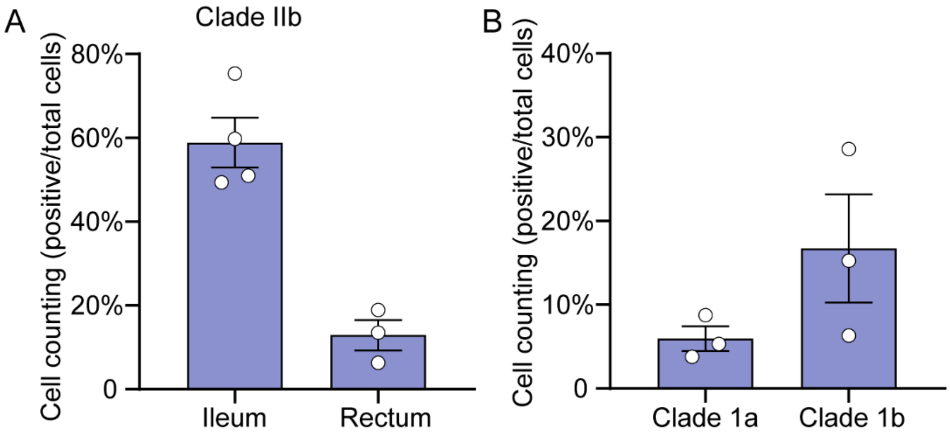

Figure S1. Quantification of MPXV-infected cells in intestinal organoids.
(A) Proportion of MPXV-positive cells relative to total cells in ileum and rectum organoids determined by immunofluorescence staining. Related to Fig. 2A. (B) Quantification of MPXV-positive cells in intestinal organoids infected with Clade 1a or Clade 1b MPXV strains. Related to Fig.7C and 7E.

Figure S2.

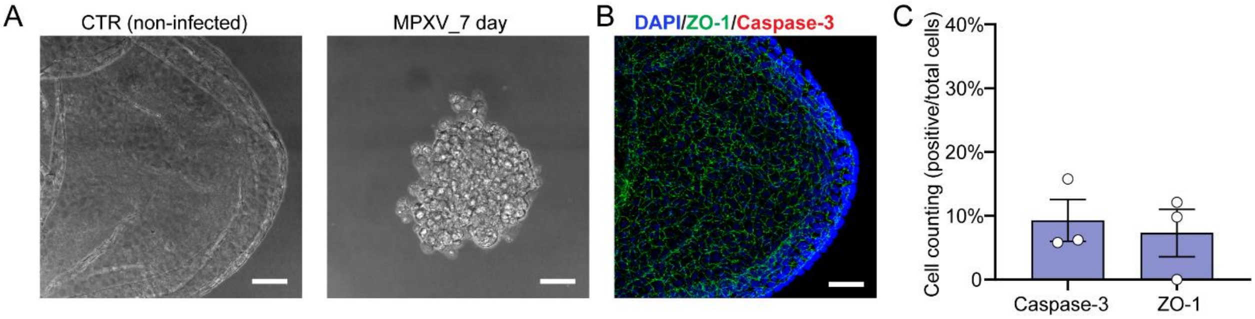

Figure S2. Morphology of MPXV infection and cell counting in intestinal organoids.
(A) Bright field images of non-infected organoids and MPXV-infected organoids after 7 days of infection. (B) Immunostaining of non-infected organoids (DAPI, nucleus; ZO-1, tight junction; Caspase-3, cell death). (C) Quantification of caspase 3-positive cells or ZO-1-positive cells relative to total cells in MPXV-infected intestinal organoids. Related to Fig. 2G. Scale bar = 50 µm.

Figure S3

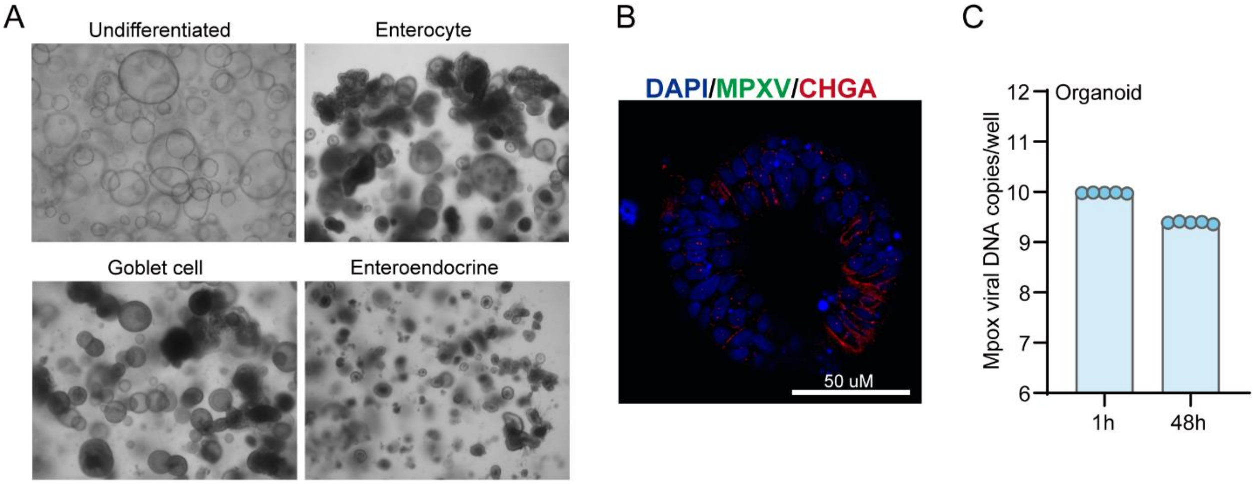

Figure S3. Modelling MPXV infection in differentiated intestinal organoids.
(A) Morphology of organoids differentiating towards enterocytes-, goblet- and enteroendocrine (EEC)-phenotypes after 5-days differentiation culture. Immunostaining MPXV virions at 48 hours post-infection (B) and QRT-PCR quantification of viral DNA level in enteroendocrine-differentiated organoids (C). CHGA (red) is the representative marker of enteroendocrine cells. MPXV (green) florescence signal was not detected in CHGA-positive organoid cells.

Figure S4

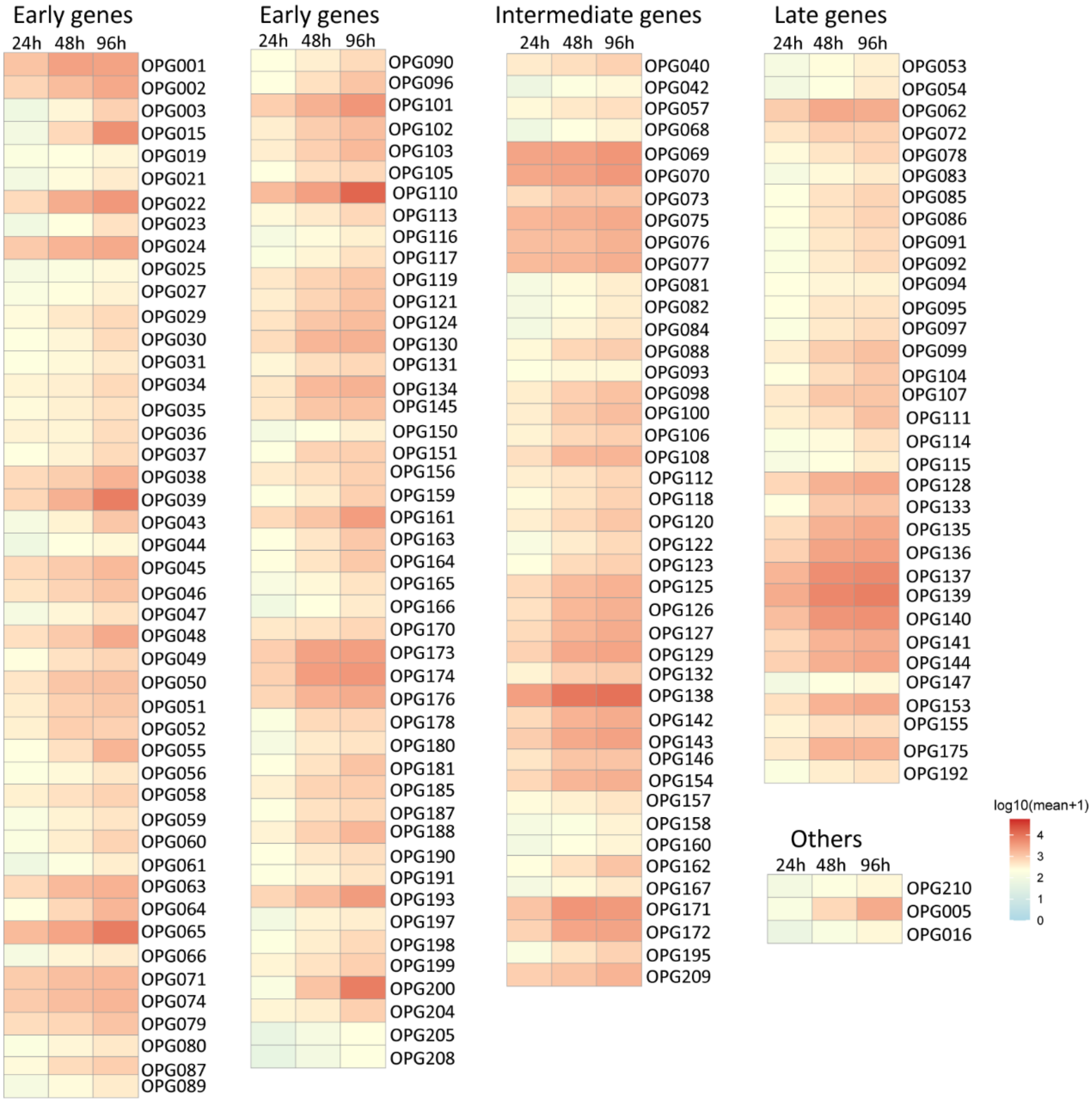

Figure S4. Heat map mapping viral transcripts at different time points post-inoculation. These transcripts are categorized according to defined early, intermediate and late genes (*Deng, et al. mBio. 2025 Apr 9;16(4):e0380924*), as well as others that are not defined. Some transcripts encoding key viral proteins markedly expressed after 48 hours-post infection, such as OPG015 (Ankyrin repeat protein), OPG022 (Interleukin-18-binding protein), OPG039 (Ankyrin-like protein), OPG065 (Double-stranded RNA binding protein), OPG101 (Thymidine kinase), OPG110 (Late transcription factor VLTF-4), OPG138 (A12 protein), OPG200 (Bcl-2-like protein).

Figure S5

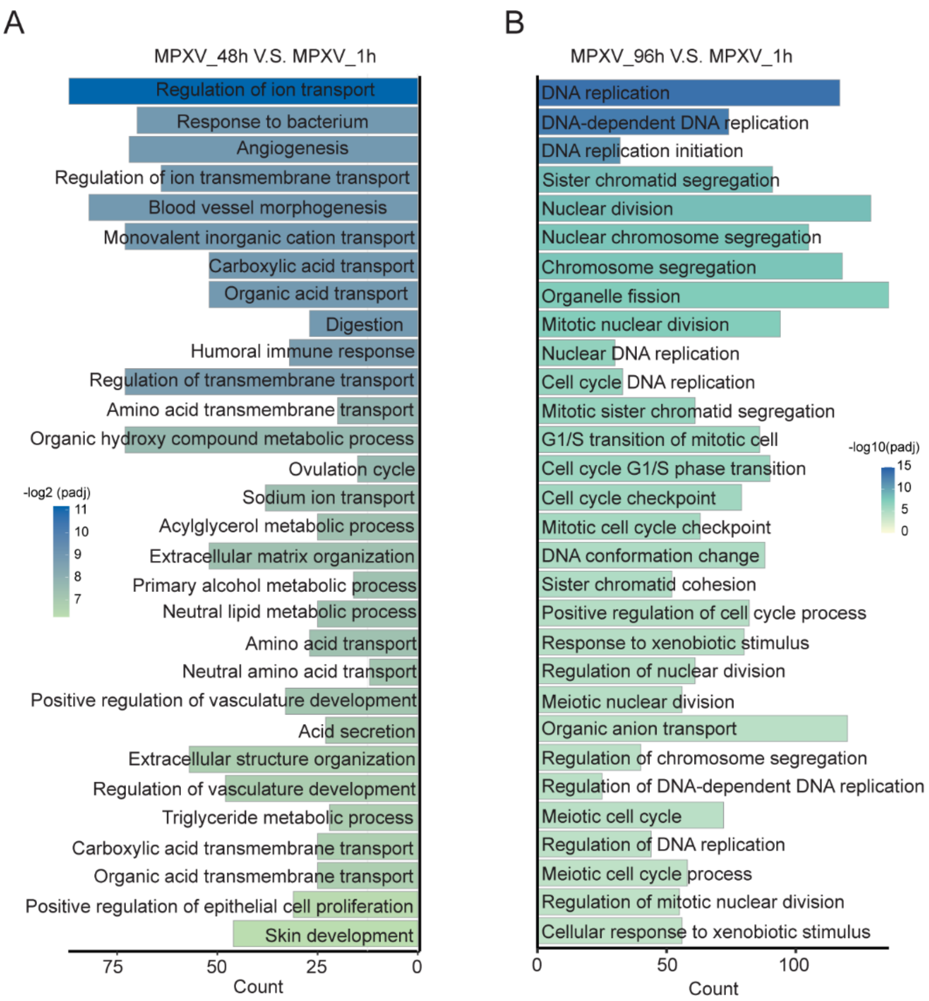

Figure S5. Gene ontology (GO) analysis of MPXV regulated pathways.
Top 30 significantly enriched pathways by GO analysis of MPXV infected organoids at 48 hours (A) and 96 hours (B), compared to 1 hour post-infection.

Figure S6

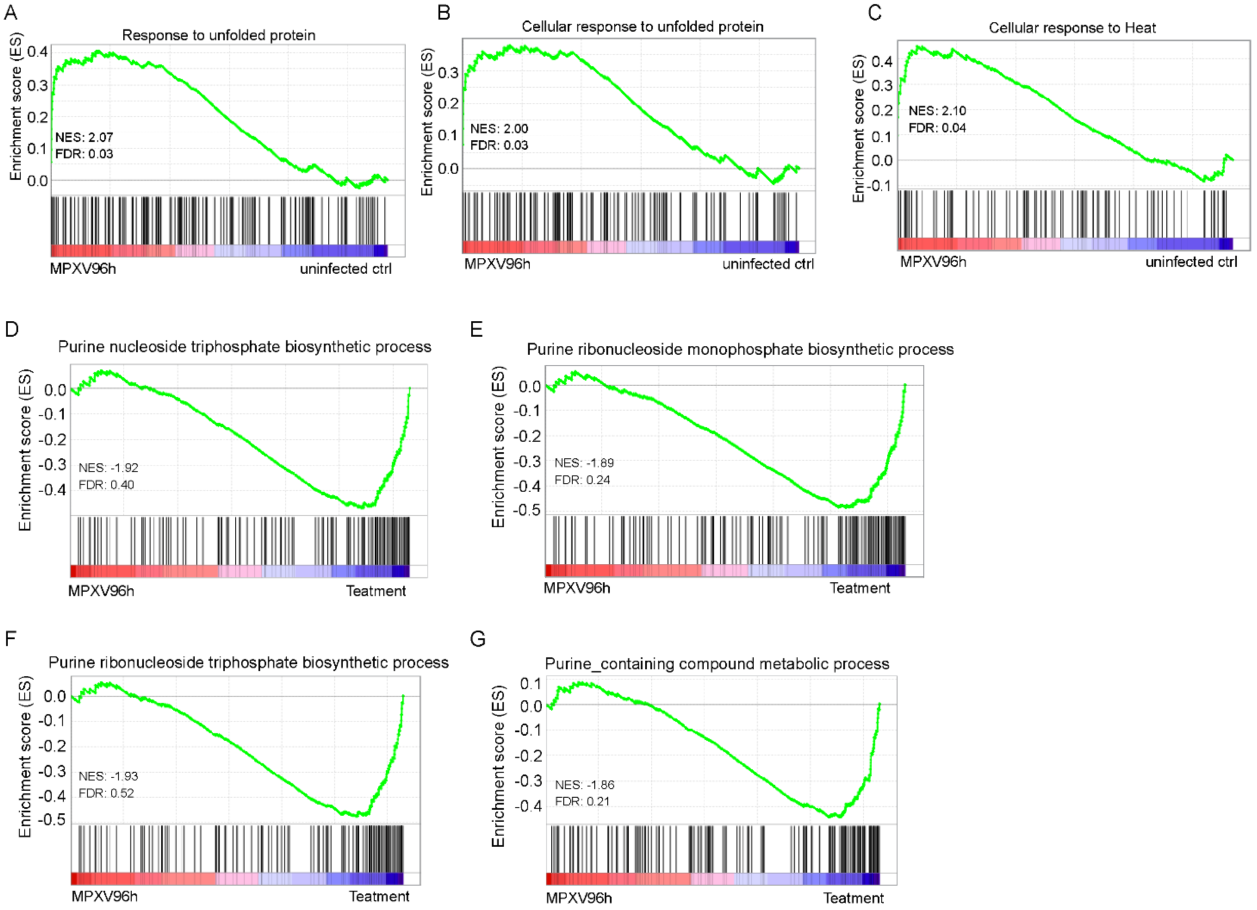

Figure S6. Gene set enrichment analysis (GSEA) of GO pathways in intestinal organoids infected with MPXV.

(A-C) Enriched transcriptional signatures in organoids of 96 h.p.i comparison with uninfected organoids. (D-G) Comparison of MPXV infected organoids for 96 hours with and without clofarabine treatment showed that the transcriptional signatures of purine nucleoside/ribonucleoside associated process were inhibited.

Figure S7

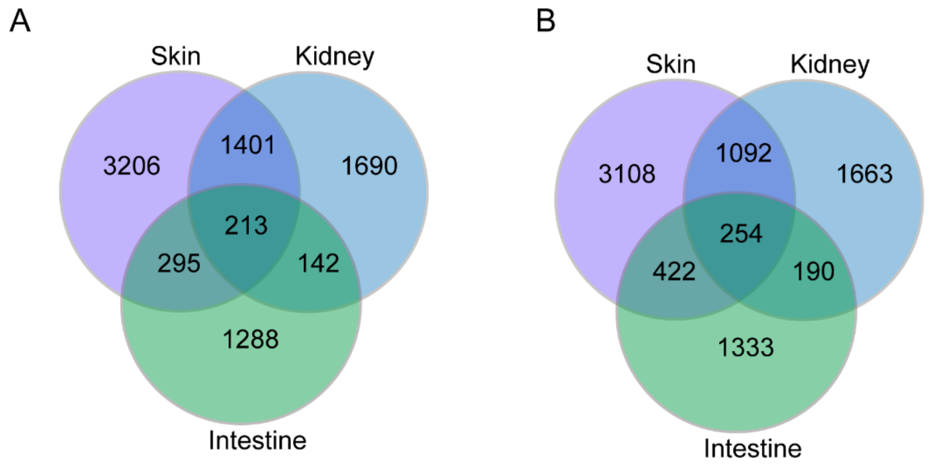

Figure S7. Comparison of transcriptomic alterations upon MPXV infections in human skin, kidney, and intestinal organoids.

Venn plot indicating the commonly upregulated (A) and downregulated genes (B) among three types of organoids triggered by MPXV infections.

Figure S8

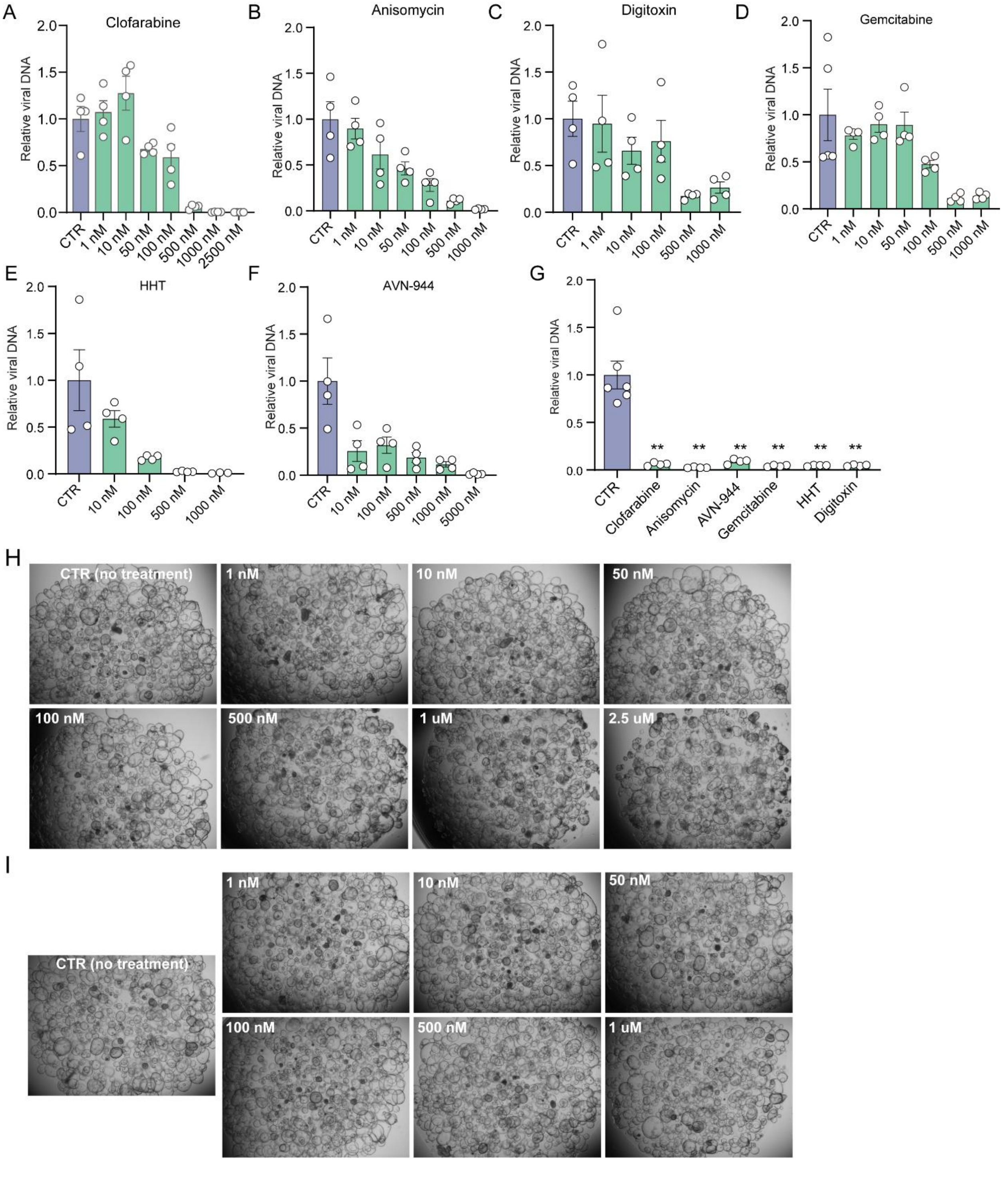

Figure S8. The antiviral effects of identified leading compounds in intestinal organoids. These include clofarabine (A), anisomycin (B), digitoxin (C), gemcitabine (D), homoharringtonine (HHT) (E), and AVN-944 (F) in MPXV-infected intestinal (ileum) organoids after 48 hours treatment. (G) The antiviral effects of tested compounds in human rectum organoids infected

with MPXV and treated for 48 hours. Bright field images of intestinal (ileum) organoids treated with different concentrations of clofarabine (H) and anisomycin (I) for 48 hours.

Figure S9.

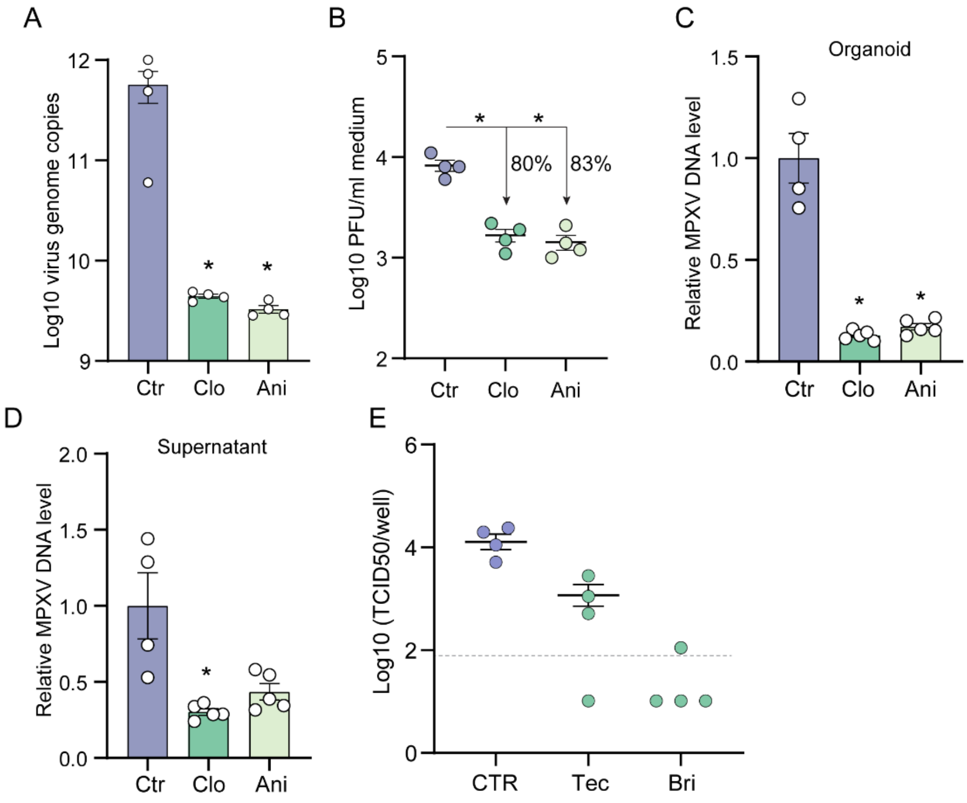

Figure S9. Validation of the antiviral activity of the leading candidates and the controls.
(A) The inhibition of clofarabine (Clo) and anisomycin (Ani) on intracellular MPXV DNA levels in intestinal organoids at the scenario of delayed treatment (the treatment initiated at 3 d.p.i). (B) Quantification of MPXV infectious titers in culture medium by 48 hours treatment in the scenario of delayed treatment (n= 4).Quantification of MPXV DNA level in organoids (C) and culture medium (D) after 7 days treatment with clofarabine (Clo) and anisomycin (Ani). (E) Quantification of intracellular infectious virus titers in MPXV-infected organoids upon tecovirimat (Tec; 1 uM) and brincidofovir (Bri; 1 uM) treatment for 48 hours.

Figure S10

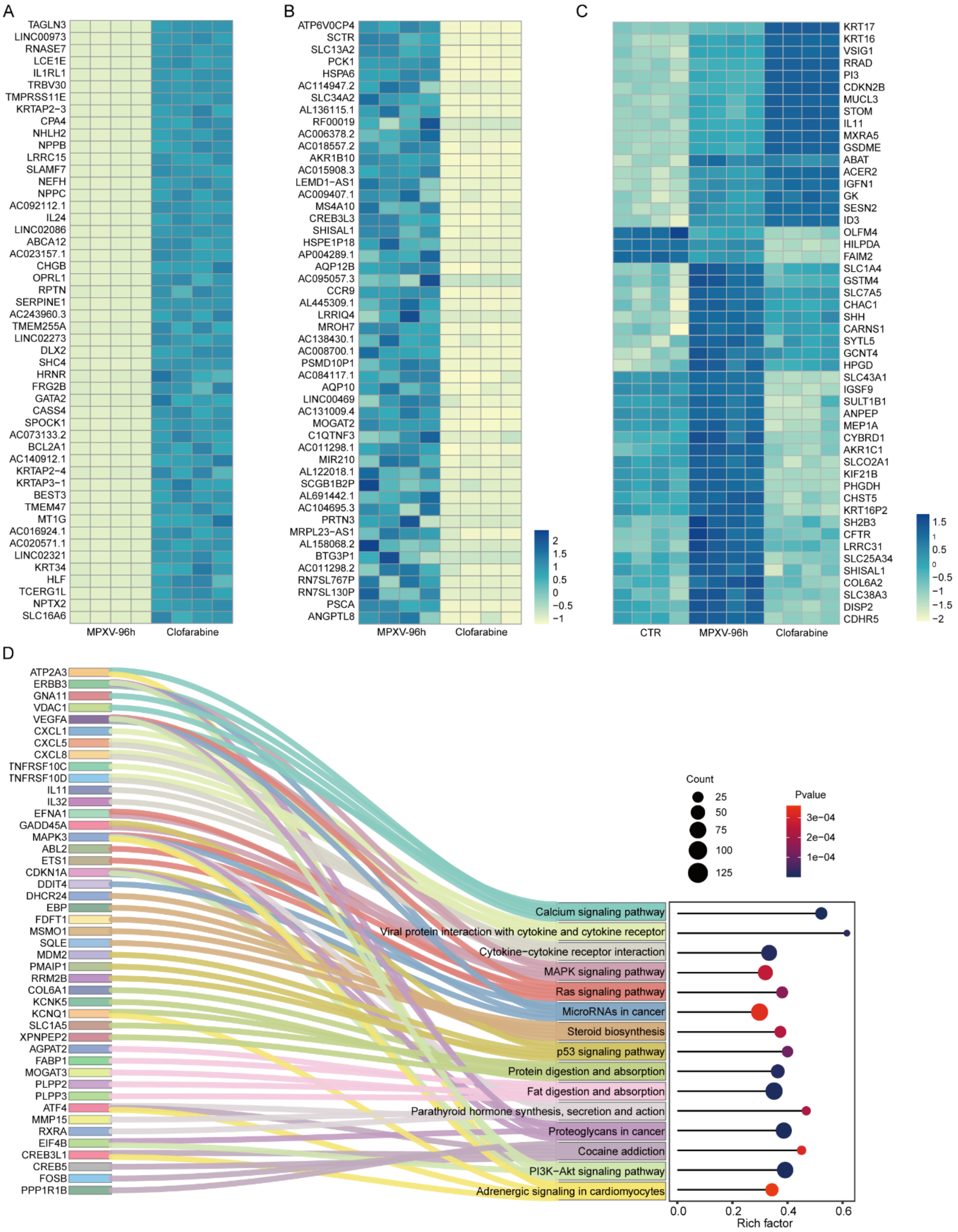

Figure S10. Significantly regulated genes and signaling pathways in intestinal organoids upon MPXV infection and clofarabine treatment.

Top 50 significantly upregulated (A) and downregulated (B) genes upon 1 µM of clofarabine treatment in MPXV-infected organoids for 96 hours. (C) Significantly regulated genes (top 51-100) upon MPXV infection for 96 hours. (D) Sankey diagram illustrates the relationship between significantly altered genes (left) and enriched KEGG pathways (right).

Figure S11

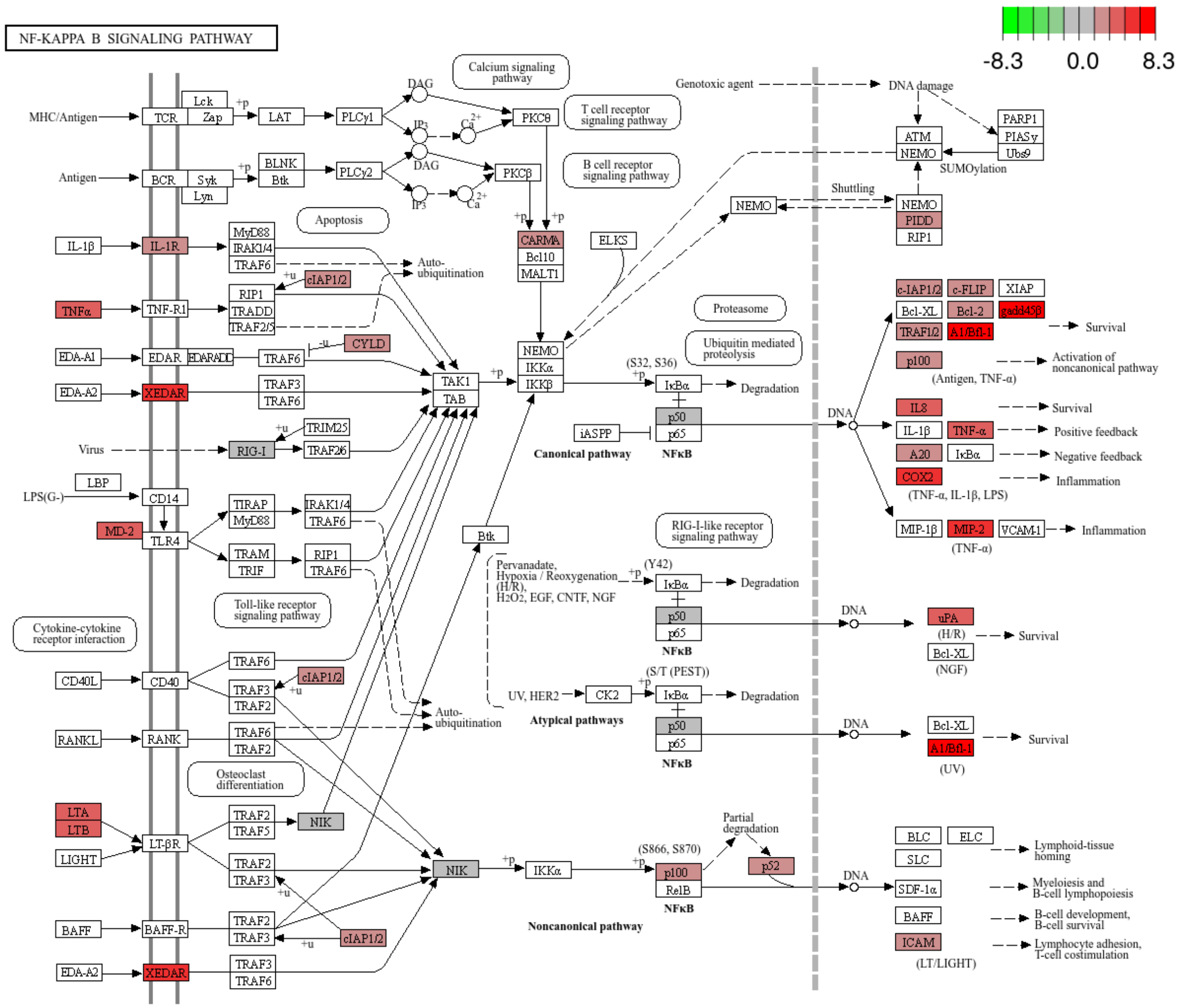

Figure S11. Gene interaction network of the NF-κB signaling pathway.

Genes shown in red are significantly upregulated in clofarabine-treated organoids compared with untreated controls at 96 hours post-infection.

Figure S12

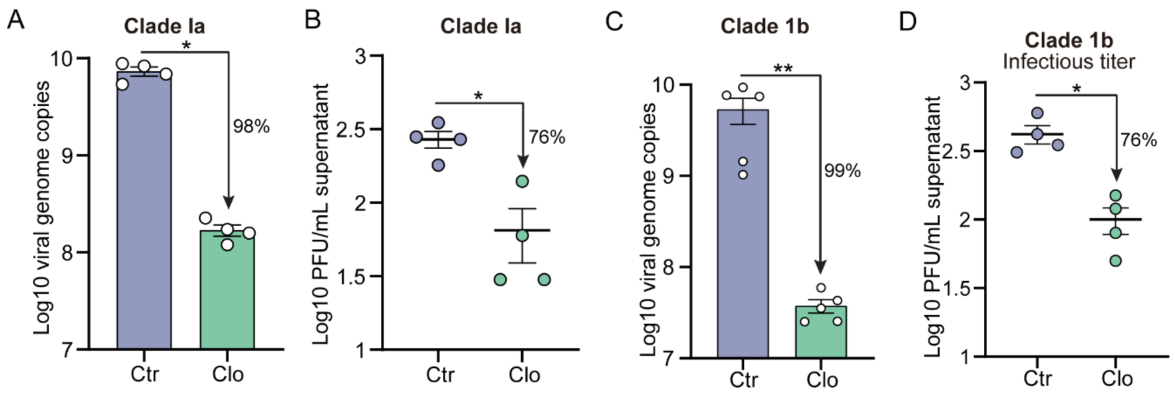

Figure S12. Antiviral effects of clofarabine in clade Ia and Ib MPXV infected intestinal organoids. (A) Quantification of the intracellular DNA level in intestinal organoids infected with clade Ia MPXV at 48 h.p.i (n = 4). (B) Quantification of the infectious virus titers in culture medium of clade Ia infected intestinal organoids at 48 h.p.i (n = 4). (C) Quantification of the viral DNA level in intestinal organoids infected with clade Ib MPXV at 48 h.p.i (n= 5). (D) Quantification of the infectious virus titers in culture medium of clade Ib infected intestinal organoids at 48 h.p.i (n = 4). Data are presented as mean ± SEM. Statistical analysis was performed using the two-tailed Mann–Whitney test. *$P < 0.05$, **$P < 0.01$.

Table S1. Primers used in this study.

Gene	Sequence (5'-3')
GAPDH-F	GTCTCCTCTGACTTCAACAGCG
GAPDH-R	ACCACCCTGTTGCTGTAGCCAA
Villin-F	GCTGCTCTACACCTACCTCATC
Villin-R	TTCTGGTCCAGGATGACGGCTT
Muc-2 F	ACTCTCCACACCCAGCATCATC
Muc-2 R	GTGTCTCCGTATGTGCCGTTGT
hCHGA-F	TGACCTCAACGATGCATTTC
hCHGA-R	CTGTCCTGGCTCTTCTGCTC
MPXV-F	GGCTCTTCTATCAACCACA
MPXV-R	AGTCATTATCTCCTCCTCCA

Table S2. Antibodies used in this study.

Antibody	Company & Catalogue number
Rabbit polyclonal anti-Vaccinia virus Lister Strain (FITC)	Abbexa, abx023199
Rabbit polyclonal anti-Vaccinia virus Lister Strain	Abbexa, abx023200
Anti-CDX2 antibody, rabbit	Abcam, AB76541
Anti-SOX9 antibody, rabbit	Abcam, AB185966
Anti-Ki67 antibody, rabbit	Abcam, AB15580
Anti-ZO-1 antibody,	Life technologies, MA339100A488
Anti-EpCAM, rabbit	Abcam, ab71916
Anti-Active Caspase-3 antibody, mouse	Bio-techne, AF835
Anti-Villin, mouse	Santa Cruz Biotechnology, sc-66022
Anti-Muc2, mouse	Santa Cruz Biotechnology, sc-59859
Anti-CHGA, mouse	Santa Cruz Biotechnology, sc-393941
Alexa Fluor 488 Goat anti-Rabbit antibody	ThermoFisher Scientific, A32731
Alexa Fluor 594 Goat anti-Mouse antibody	ThermoFisher Scientific, A32742

Table S3. Overlapped upregulated genes among skin, kidney, and intestinal organoids upon MPXV infections.

HSPA1A	MALAT1	AC108134.2	HMOX1	EEF1A2	KRT16P2	IRS3P	C2CD2L	SYT7	GUCA1B
HSPH1	AC131212.3	LINC00115	C6orf141	PTPRH	AC131009.4	MIR1249	CD55	TMEM92-AS1	CARS-AS1
HSPA6	AP003469.4	BHLHE40	COX6B2	HSPB1	AL137003.2	HLA-K	AC068580.1	RN7SL472P	HCG4B
HIST1H1E	CLDND1	AP000648.3	GSDMB	HLA-G	AL021807.1	OGFR-AS1	AC124944.3	TNFSF13B	ERN1
HSPA1B	IGFN1	BX284668.2	NEAT1	STX11	IRF9	AC105429.1	AC005479.1	AL391244.3	KBTBD11-OT1
PHF1	INSIG1	KRT16	HIST1H2BC	SDHAP1	PIM1	HK2	ACHE	ZNF451-AS1	AL391684.1
IL11	ZFAND2A	HIST1H2BD	PRKCD	AC004264.1	NPM1P26	AC011472.1	AC125421.1	SGSM3	HSPB8
STX1A	HIST1H1C	FAM214B	AL390719.1	AC005253.1	MICA	ARL6IP4	BX255925.1	SLPI	AP002364.1
ARC	HOTAIRM1	GNRH1	LAMB3	HIST1H4C	AC132008.2	SUMO4	HCN2	AL133230.1	DUOX2
DUSP5	HIST1H2BJ	IL6R	LINC01783	HIST1H3PS1	AURKC	TBC1D10A	RF00019	AL049840.1	GIPR
VGF	LSMEM1	MXD1	RNF103-CHMP3	AC013394.1	GOLGA7B	ISG20	AC008764.8	PDP2	TBX19
DDIT4	AC124068.2	MIR222HG	DNAJA4	LINC01004	RIOK3	NIPA1	RUNDC3B	FAM126B	AC004908.1
RSRP1	AKR1C1	CAPN10-DT	MKNK2	AC004908.2	C17orf67	AP001052.1	LAMC2	ABHD11-AS1	AC243965.2
ICAM5	AC132872.1	HIST2H2BE	KCMF1	UNC5B-AS1	AL021918.3	AC103706.1	PLAC8	AL731533.2	AC005326.1
RN7SL3	AL158152.2	FTL	KLKP1	AC110285.6	KIF1A	SHISA8	LGALS7B	CBX3P4	TMEM54
AC232271.1	BRSK2	CCDC88B	ZNF436-AS1	FER1L4	ZC3H12D	MIR29B2CHG	AC025287.3	AC008687.4	
AC015853.1	AL662844.4	HIST1H3E	AC004477.1	PPP2R2C	TCP11L2	HCG27	LINC00452	EDN2	
AC091185.1	AC068580.3	PIWIL2	AC063976.2	URAHP	CEP295NL	AC233968.1	ASIC4	BLACAT1	
AL031717.1	AC139887.2	SPAG4	LINC00469	S100A6	AL121832.3	AC006378.2	SULT1A3	AP000350.2	
OSTM1	SH2D6	KRT16P1	AP004289.1	AL133367.1	PROCR	AC025857.2	ITGB6	CPT1B	
BTG3P1	HCFC2	AL442125.1	UBE2Q1-AS1	AC005921.3	DSG2-AS1	PICART1	SIGLEC15	ST13P12	
AC002553.1	PANX2	SLC7A11-AS1	GDPD3	AC010618.3	SLC6A20	KCNK6	FNDC11	AC145207.9	

Table S4. Overlapped downregulated genes among skin, kidney, and intestinal organoids upon MPXV infections.

ADGRA2	CNTNAP2	NFIA	DLC1	TYMS	LMX1B	LINC01963	FAM229B	CAVIN2	PRIM1
FOXRED2	ZNF423	CHST10	TROAP	RAB36	PKMYT1	STOX1	STK33	KATNAL2	NEIL3
RGL1	C1QTNF2	ZWINT	CDCA5	KIF4A	PART1	UBXN11	FGD3	BLM	CDCA8
SCARA3	ROR1	DAPL1	GDF11	HJURP	SH3PXD2B	PCLAF	RAD51AP1	CDKN3	RARRES2
SLC16A10	TMEM150C	LMNB1	ST6GALNAC3	CPVL	KDELC1	CENPA	SKA1	NME5	TENT5C
PDZRN3	CIT	KIFC1	CHAF1A	IQGAP3	BUB1B	IFI6	CCDC34	DNER	CCDC180
PRMT6	FOXM1	NBN	RNF144A	RRM2	UCP2	GINS2	CDC25C	DDIAS	ORC1
TOP2A	DPYSL3	CACHD1	DLGAP5	NCAPH	MAP6	TMPO-AS1	NUF2	ARHGAP24	SUSD2
TCF4	PLXNA4	ANXA6	PHYHD1	GPC4	RMDN2	SKA3	PRCP	HMMR	FANCD2
FNDC10	SETMAR	ASPM	CNTLN	MCM5	PCDH7	FAHD2B	SLC16A11	PRKCG	AC004540.1
EXTL2	FBN3	STMN1	TNFRSF13C	ZBTB12	DHFRP1	GINS1	SPC25	TSNAXIP1	ITGB3BP
POLR3H	TOX	SAPCD2	LINC00680	AC091057.1	CCDC138	MCM4	APLN	MYB	SGO2
MKI67	OSR2	MYBL2	SMO	CDK1	PCNA	SPC24	CCNA2	OXGR1	NRGN
NREP	FBLN5	MFAP2	E2F1	CENPE	RMI2	MID1	NCAPG	AURKA	LAMA2
ADAMTS15	ZNF300	KIF2C	GTSE1	NTRK2	CD83	SIAH3	LRRTM1	REXO5	DNAH6
TACC3	ATP6V0E2	H2AFY2	CKAP2L	PYCR3	SFXN2	MELK	CENPK	NUDT6	DSCC1
FREM2	BIRC5	LRRN1	YPEL1	DTL	IFT22	NELL1	HMGN5	TRIP13	FAM201A
CENPF	RIMS4	QDPR	DEPDC1	NDC80	FBLN1	COLGALT2	PBK	KIF23	AC007906.2
SNX29	KIF11	NKD1	CCDC80	CCNB2	AURKB	CDC45	MCM10	NEBL-AS1	ESCO2
DHFR	PPM1H	PIGZ	WWOX	KNL1	TIMELESS	SMOC1	CENPH	PDE3B	OIP5
EDAR	PTPN13	SPEF2	BRCA1	FZD2	TK1	EXO1	KCNJ2	FAM86JP	LINC00672
PARD6G	LSAMP	TNFRSF19	SP5	EFCAB11	SEMA3G	PARPBP	PIMREG	IGFBPL1	MDH1B
TCF19	CEP78	KIF20A	MYLK	FANCL	FAM111B	RASL11B	DPH6	E2F2	VASH2
ZNF618	CHADL	C5	KIF18B	TTK	ASB9	PIF1	HAUS1	AL109811.3	DNMT3B
LGR5	MCM2	DUT	HENMT1	NEK2	KIF15	BUB1	BRIP1	CYP1B1-AS1	ADAMTS19-AS1
MYT1	EFCC1	APOLD1	MND1						
